# Supplementary material for: Experimental and Computational Investigation of the Oxime Bond Stereochemistry in c-Jun N-terminal Kinase 3 Inhibitors 11H-Indeno[1,2-b]quinoxalin-11-one Oxime and Tryptanthrin-6-oxime
Source: Pharmaceutics. 2023 Jun 23;15(7):1802. doi: 10.3390/pharmaceutics15071802 (PMC10383563; doi:10.3390/pharmaceutics15071802)

checkCIF/PLATON report

Structure factors have been supplied for datablock(s) vvm-trfin_a

THIS REPORT IS FOR GUIDANCE ONLY. IF USED AS PART OF A REVIEW PROCEDURE FOR PUBLICATION, IT SHOULD NOT REPLACE THE EXPERTISE OF AN EXPERIENCED CRYSTALLOGRAPHIC REFEREE.

No syntax errors found.   CIF dictionary   Interpreting this report

Datablock: vvm-trfin_a

```
Bond precision:   C-C = 0.0091 A          Wavelength=0.75270

Cell:              a=13.614(3)      b=4.898(1)          c=17.322(4)
                   alpha=90         beta=90.12(3)       gamma=90
Temperature:       100 K

                 Calculated                    Reported
Volume           1155.1(4)                     1155.1(4)
Space group      P 21                          P 21
Hall group       P 2yb                         P 2yb
Moiety formula   C15 H9 N3 O2                  ?
Sum formula      C15 H9 N3 O2                  C15 H9 N3 O2
Mr               263.25                        263.25
Dx,g cm-3        1.514                         1.514
Z                4                             4
Mu (mm-1)        0.117                         0.117
F000             544.0                         544.0
F000'            544.29
h,k,lmax         16,5,21                       16,5,21
Nref             4367[ 2459]                   4249
Tmin,Tmax        0.999,0.999                   0.805,1.000
Tmin'            0.983

Correction method= # Reported T Limits: Tmin=0.805 Tmax=1.000
AbsCorr = MULTI-SCAN

Data completeness= 1.73/0.97        Theta(max)= 27.318

R(reflections)= 0.0659( 3075)                        wR2(reflections)=
                                                     0.1708( 4249)
S = 1.029                    Npar= 542
```

The following ALERTS were generated. Each ALERT has the format
test-name_ALERT_alert-type_alert-level.
Click on the hyperlinks for more details of the test.

Alert level B

```
PLAT089_ALERT_3_B Poor Data / Parameter Ratio (Zmax < 18) ........       4.51 Note
```

Alert level C

```
DIFMX02_ALERT_1_C  The maximum difference density is > 0.1*ZMAX*0.75
            The relevant atom site should be identified.
STRVA01_ALERT_4_C           Flack parameter is too small
           From the CIF: _refine_ls_abs_structure_Flack   -1.300
           From the CIF: _refine_ls_abs_structure_Flack_su    1.000
PLAT094_ALERT_2_C Ratio of Maximum / Minimum Residual Density ....       2.57 Report
PLAT097_ALERT_2_C Large Reported Max.  (Positive) Residual Density       0.61 eA-3
PLAT340_ALERT_3_C Low Bond Precision on  C-C Bonds ..............    0.00913 Ang.
PLAT906_ALERT_3_C Large K Value in the Analysis of Variance ......      2.672 Check
PLAT911_ALERT_3_C Missing FCF Refl Between Thmin & STh/L=    0.600         16 Report
```

Alert level G

```
ABSMU01_ALERT_1_G  Calculation of _exptl_absorpt_correction_mu
                not performed for this radiation type.
PLAT002_ALERT_2_G Number of Distance or Angle Restraints on AtSite         40 Note
PLAT003_ALERT_2_G Number of Uiso or Uij Restrained non-H Atoms ...         40 Report
PLAT007_ALERT_5_G Number of Unrefined Donor-H Atoms .............           3 Report
PLAT032_ALERT_4_G Std. Uncertainty on Flack Parameter Value High .      1.000 Report
PLAT092_ALERT_4_G Check: Wavelength Given is not Cu,Ga,Mo,Ag,In Ka    0.75270 Ang.
PLAT176_ALERT_4_G The CIF-Embedded .res File Contains SADI Records          8 Report
PLAT178_ALERT_4_G The CIF-Embedded .res File Contains SIMU Records          1 Report
PLAT188_ALERT_3_G A Non-default SIMU Restraint Value has been used     0.0100 Report
PLAT191_ALERT_3_G A Non-default SADI Restraint Value has been used     0.0100 Report
PLAT191_ALERT_3_G A Non-default SADI Restraint Value has been used     0.0100 Report
PLAT191_ALERT_3_G A Non-default SADI Restraint Value has been used     0.0100 Report
PLAT191_ALERT_3_G A Non-default SADI Restraint Value has been used     0.0100 Report
PLAT191_ALERT_3_G A Non-default SADI Restraint Value has been used     0.0100 Report
PLAT191_ALERT_3_G A Non-default SADI Restraint Value has been used     0.0100 Report
PLAT191_ALERT_3_G A Non-default SADI Restraint Value has been used     0.0100 Report
PLAT191_ALERT_3_G A Non-default SADI Restraint Value has been used     0.0100 Report
PLAT301_ALERT_3_G Main Residue  Disorder ..............(Resd  2  )       100% Note
PLAT301_ALERT_3_G Main Residue  Disorder ..............(Resd  3  )       100% Note
PLAT304_ALERT_4_G Non-Integer Number of Atoms in ..... (Resd  2  )      18.76 Check
PLAT304_ALERT_4_G Non-Integer Number of Atoms in ..... (Resd  3  )      10.24 Check
PLAT720_ALERT_4_G Number of Unusual/Non-Standard Labels ..........          2 Note
PLAT811_ALERT_5_G No ADDSYM Analysis: Too Many Excluded Atoms ....          ! Info
PLAT860_ALERT_3_G Number of Least-Squares Restraints .............       1075 Note
PLAT916_ALERT_2_G Hooft y and Flack x Parameter Values Differ by .       1.10 Check
PLAT933_ALERT_2_G Number of HKL-OMIT Records in Embedded .res File          4 Note
PLAT941_ALERT_3_G Average HKL Measurement Multiplicity ...........        2.9 Low
PLAT961_ALERT_5_G Dataset Contains no Negative Intensities .......     Please Check
PLAT978_ALERT_2_G Number C-C Bonds with Positive Residual Density.          1 Info
```

```
 0 ALERT level A = Most likely a serious problem - resolve or explain
 1 ALERT level B = A potentially serious problem, consider carefully
 7 ALERT level C = Check. Ensure it is not caused by an omission or oversight
29 ALERT level G = General information/check it is not something unexpected

 2 ALERT type 1 CIF construction/syntax error, inconsistent or missing data
 7 ALERT type 2 Indicator that the structure model may be wrong or deficient
17 ALERT type 3 Indicator that the structure quality may be low
 8 ALERT type 4 Improvement, methodology, query or suggestion
 3 ALERT type 5 Informative message, check
```

It is advisable to attempt to resolve as many as possible of the alerts in all categories. Often the minor alerts point to easily fixed oversights, errors and omissions in your CIF or refinement strategy, so attention to these fine details can be worthwhile. In order to resolve some of the more serious problems it may be necessary to carry out additional measurements or structure refinements. However, the purpose of your study may justify the reported deviations and the more serious of these should normally be commented upon in the discussion or experimental section of a paper or in the "special_details" fields of the CIF. checkCIF was carefully designed to identify outliers and unusual parameters, but every test has its limitations and alerts that are not important in a particular case may appear. Conversely, the absence of alerts does not guarantee there are no aspects of the results needing attention. It is up to the individual to critically assess their own results and, if necessary, seek expert advice.

Publication of your CIF in IUCr journals

A basic structural check has been run on your CIF. These basic checks will be run on all CIFs submitted for publication in IUCr journals (*Acta Crystallographica*, *Journal of Applied Crystallography*, *Journal of Synchrotron Radiation*); however, if you intend to submit to *Acta Crystallographica Section C* or *E* or *IUCrData*, you should make sure that full publication checks are run on the final version of your CIF prior to submission.

Publication of your CIF in other journals

Please refer to the *Notes for Authors* of the relevant journal for any special instructions relating to CIF submission.

PLATON version of 28/11/2022; check.def file version of 28/11/2022

Datablock vvm-trfin_a - ellipsoid plot

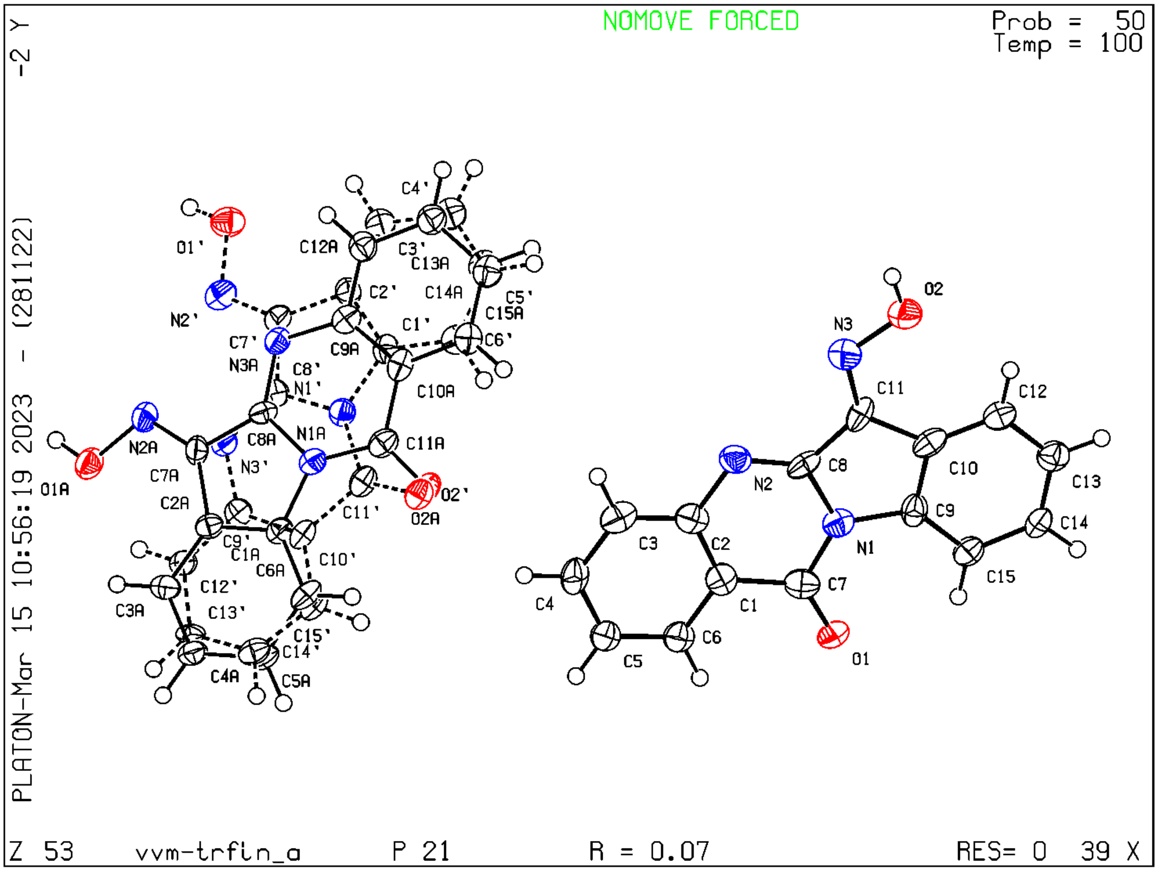

Supplement: Supplementary file 1 [file pharmaceutics-15-01802-s001.zip › 2249116_CheckCIF.pdf]
