# Supplementary material for: Experimental and Computational Investigation of the Oxime Bond Stereochemistry in c-Jun N-terminal Kinase 3 Inhibitors 11H-Indeno[1,2-b]quinoxalin-11-one Oxime and Tryptanthrin-6-oxime
Source: Pharmaceutics. 2023 Jun 23;15(7):1802. doi: 10.3390/pharmaceutics15071802 (PMC10383563; doi:10.3390/pharmaceutics15071802)

checkCIF/PLATON report

Structure factors have been supplied for datablock(s) iq1a_mi_a_sq

THIS REPORT IS FOR GUIDANCE ONLY. IF USED AS PART OF A REVIEW PROCEDURE FOR PUBLICATION, IT SHOULD NOT REPLACE THE EXPERTISE OF AN EXPERIENCED CRYSTALLOGRAPHIC REFEREE.

No syntax errors found. CIF dictionary Interpreting this report

Datablock: iq1a_mi_a_sq

```
Bond precision:   C-C = 0.0041 A          Wavelength=1.54178

Cell:              a=4.4961(1)     b=23.1804(4)       c=14.3978(3)
                   alpha=90        beta=98.812(1)     gamma=90
Temperature:       150 K

                  Calculated                         Reported
Volume            1482.85(5)                         1482.85(5)
Space group       I a                                I a
Hall group        I -2ya                             I -2ya
Moiety formula    C15 H9 N3 O [+ solvent]            ?
Sum formula       C15 H9 N3 O [+ solvent]            C15 H9 N3 O
Mr                247.25                             247.26
Dx,g cm-3         1.107                              1.108
Z                 4                                  4
Mu (mm-1)         0.587                              0.587
F000              512.0                              512.0
F000'             513.54
h,k,lmax          5,27,17                            5,27,17
Nref              2629[ 1321]                        2054
Tmin,Tmax         0.932,0.971                        0.689,0.753
Tmin'             0.905

Correction method= # Reported T Limits: Tmin=0.689 Tmax=0.753
AbsCorr = MULTI-SCAN

Data completeness= 1.55/0.78        Theta(max)= 66.438

R(reflections)= 0.0368( 1983)                         wR2(reflections)=
                                                      0.1062( 2054)

S = 1.061                    Npar= 175
```

The following ALERTS were generated. Each ALERT has the format
test-name_ALERT_alert-type_alert-level.
Click on the hyperlinks for more details of the test.

Alert level C

```
PLAT340_ALERT_3_C Low Bond Precision on  C-C Bonds ...............    0.00406 Ang.
PLAT911_ALERT_3_C Missing FCF Refl Between Thmin & STh/L=    0.595          3 Report
```

Alert level G

```
PLAT605_ALERT_4_G Largest Solvent Accessible VOID in the Structure        205 A**3
PLAT869_ALERT_4_G ALERTS Related to the Use of SQUEEZE  Suppressed          ! Info
PLAT909_ALERT_3_G Percentage of I>2sig(I) Data at Theta(Max) Still        94% Note
PLAT915_ALERT_3_G No Flack x Check Done: Low Friedel Pair Coverage         56 %
PLAT916_ALERT_2_G Hooft y and Flack x Parameter Values Differ by .       0.14 Check
PLAT941_ALERT_3_G Average HKL Measurement Multiplicity ...........        3.2 Low
PLAT961_ALERT_5_G Dataset Contains no Negative Intensities .......     Please Check
PLAT978_ALERT_2_G Number C-C Bonds with Positive Residual Density.          0 Info
```

0 **ALERT level A** = Most likely a serious problem - resolve or explain
0 **ALERT level B** = A potentially serious problem, consider carefully
2 **ALERT level C** = Check. Ensure it is not caused by an omission or oversight
8 **ALERT level G** = General information/check it is not something unexpected

0 ALERT type 1 CIF construction/syntax error, inconsistent or missing data
2 ALERT type 2 Indicator that the structure model may be wrong or deficient
5 ALERT type 3 Indicator that the structure quality may be low
2 ALERT type 4 Improvement, methodology, query or suggestion
1 ALERT type 5 Informative message, check

It is advisable to attempt to resolve as many as possible of the alerts in all categories. Often the minor alerts point to easily fixed oversights, errors and omissions in your CIF or refinement strategy, so attention to these fine details can be worthwhile. In order to resolve some of the more serious problems it may be necessary to carry out additional measurements or structure refinements. However, the purpose of your study may justify the reported deviations and the more serious of these should normally be commented upon in the discussion or experimental section of a paper or in the "special_details" fields of the CIF. checkCIF was carefully designed to identify outliers and unusual parameters, but every test has its limitations and alerts that are not important in a particular case may appear. Conversely, the absence of alerts does not guarantee there are no aspects of the results needing attention. It is up to the individual to critically assess their own results and, if necessary, seek expert advice.

Publication of your CIF in IUCr journals

A basic structural check has been run on your CIF. These basic checks will be run on all CIFs submitted for publication in IUCr journals (*Acta Crystallographica*, *Journal of Applied Crystallography*, *Journal of Synchrotron Radiation*); however, if you intend to submit to *Acta Crystallographica Section C* or *E* or *IUCrData*, you should make sure that full publication checks are run on the final version of your CIF prior to submission.

Publication of your CIF in other journals

Please refer to the *Notes for Authors* of the relevant journal for any special instructions relating to CIF submission.

PLATON version of 28/11/2022; check.def file version of 28/11/2022

Datablock iq1a_mi_a_sq - ellipsoid plot

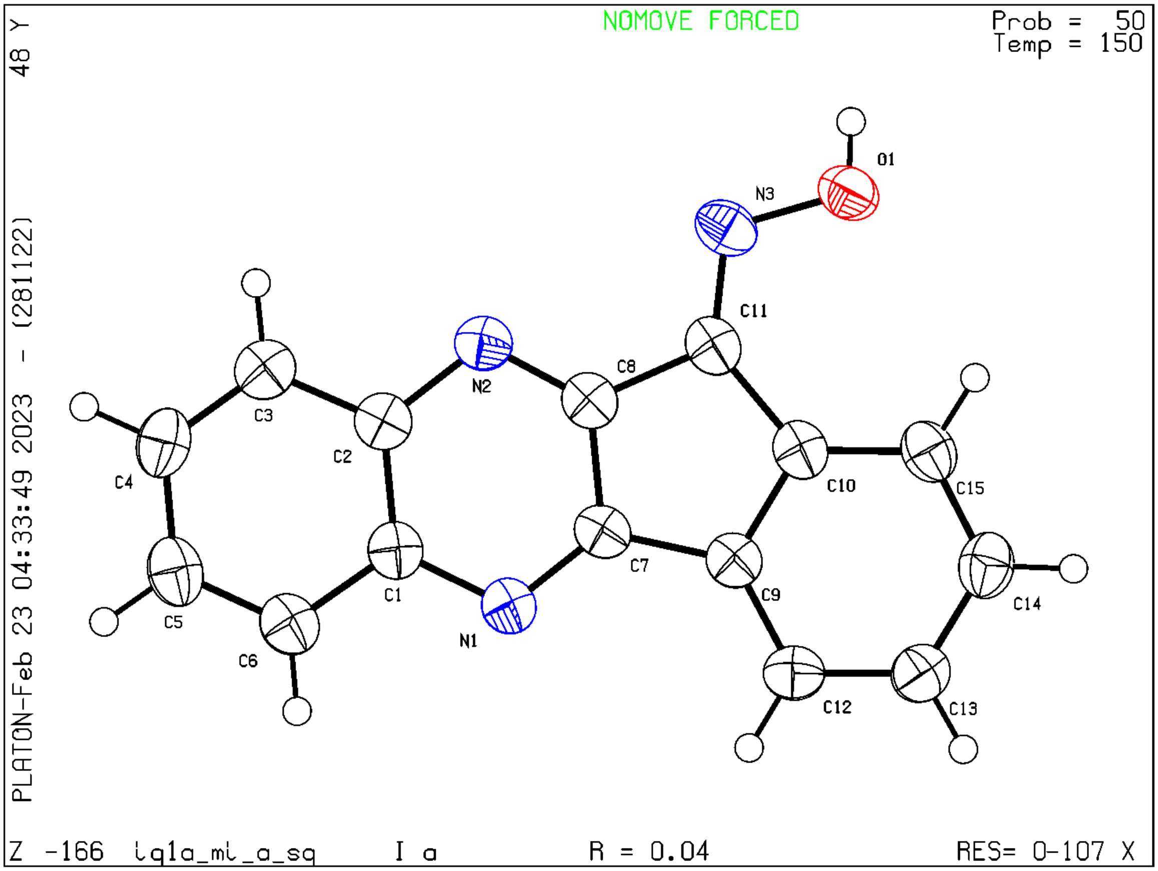

Supplement: Supplementary file 1 [file pharmaceutics-15-01802-s001.zip › 2249117_CheckCIF.pdf]
