# Supplementary material for: Experimental and Computational Investigation of the Oxime Bond Stereochemistry in c-Jun N-terminal Kinase 3 Inhibitors 11H-Indeno[1,2-b]quinoxalin-11-one Oxime and Tryptanthrin-6-oxime
Source: Pharmaceutics. 2023 Jun 23;15(7):1802. doi: 10.3390/pharmaceutics15071802 (PMC10383563; doi:10.3390/pharmaceutics15071802)

checkCIF/PLATON report

Structure factors have been supplied for datablock(s) v-trp-1

THIS REPORT IS FOR GUIDANCE ONLY. IF USED AS PART OF A REVIEW PROCEDURE FOR PUBLICATION, IT SHOULD NOT REPLACE THE EXPERTISE OF AN EXPERIENCED CRYSTALLOGRAPHIC REFEREE.

No syntax errors found. CIF dictionary Interpreting this report

Datablock: v-trp-1

```
Bond precision:   C-C = 0.0030 A          Wavelength=1.54178

Cell:              a=5.0745(1)     b=12.3974(2)        c=25.5587(4)
                   alpha=90        beta=92.126(1)      gamma=90
Temperature:       92 K

                Calculated                    Reported
Volume          1606.81(5)                    1606.81(5)
Space group     P 21/n                        P 1 21/n 1
Hall group      -P 2yn                        -P 2yn
Moiety formula  C15 H9 N3 O2, C5 H5 N         C15 H9 N3 O2, C5 H5 N
Sum formula     C20 H14 N4 O2                 C20 H14 N4 O2
Mr              342.35                        342.35
Dx,g cm-3       1.415                         1.415
Z               4                             4
Mu (mm-1)       0.774                         0.774
F000            712.0                         712.0
F000'           714.18
h,k,lmax        6,15,31                       5,15,31
Nref            3297                          3131
Tmin,Tmax       0.862,0.926                   0.688,0.754
Tmin'           0.830

Correction method= # Reported T Limits: Tmin=0.688 Tmax=0.754
AbsCorr = MULTI-SCAN

Data completeness= 0.950           Theta(max)= 74.549

R(reflections)= 0.0464( 2209)                          wR2(reflections)=
                                                       0.1251( 3131)
S = 1.026                    Npar= 238
```

The following ALERTS were generated. Each ALERT has the format
test-name_ALERT_alert-type_alert-level.
Click on the hyperlinks for more details of the test.

Alert level C

```
PLAT029_ALERT_3_C _diffrn_measured_fraction_theta_full value Low .      0.973 Why?
PLAT250_ALERT_2_C Large U3/U1 Ratio for Average U(i,j) Tensor ....        2.2 Note
PLAT355_ALERT_3_C Long   O-H (X0.82,N0.98A)  O2       - H2       .       1.03 Ang.
PLAT911_ALERT_3_C Missing FCF Refl Between Thmin & STh/L=    0.600         56 Report
```

Alert level G

```
PLAT912_ALERT_4_G Missing # of FCF Reflections Above STh/L=  0.600         28 Note
PLAT933_ALERT_2_G Number of HKL-OMIT Records in Embedded .res File          2 Note
PLAT978_ALERT_2_G Number C-C Bonds with Positive Residual Density.          8 Info
```

0 **ALERT level A** = Most likely a serious problem - resolve or explain
0 **ALERT level B** = A potentially serious problem, consider carefully
4 **ALERT level C** = Check. Ensure it is not caused by an omission or oversight
3 **ALERT level G** = General information/check it is not something unexpected

0 ALERT type 1 CIF construction/syntax error, inconsistent or missing data
3 ALERT type 2 Indicator that the structure model may be wrong or deficient
3 ALERT type 3 Indicator that the structure quality may be low
1 ALERT type 4 Improvement, methodology, query or suggestion
0 ALERT type 5 Informative message, check

It is advisable to attempt to resolve as many as possible of the alerts in all categories. Often the minor alerts point to easily fixed oversights, errors and omissions in your CIF or refinement strategy, so attention to these fine details can be worthwhile. In order to resolve some of the more serious problems it may be necessary to carry out additional measurements or structure refinements. However, the purpose of your study may justify the reported deviations and the more serious of these should normally be commented upon in the discussion or experimental section of a paper or in the "special_details" fields of the CIF. checkCIF was carefully designed to identify outliers and unusual parameters, but every test has its limitations and alerts that are not important in a particular case may appear. Conversely, the absence of alerts does not guarantee there are no aspects of the results needing attention. It is up to the individual to critically assess their own results and, if necessary, seek expert advice.

Publication of your CIF in IUCr journals

A basic structural check has been run on your CIF. These basic checks will be run on all CIFs submitted for publication in IUCr journals (*Acta Crystallographica*, *Journal of Applied Crystallography*, *Journal of Synchrotron Radiation*); however, if you intend to submit to *Acta Crystallographica Section C* or *E* or *IUCrData*, you should make sure that full publication checks are run on the final version of your CIF prior to submission.

Publication of your CIF in other journals

Please refer to the *Notes for Authors* of the relevant journal for any special instructions relating to CIF submission.

PLATON version of 28/11/2022; check.def file version of 28/11/2022

Datablock v-trp-1 - ellipsoid plot

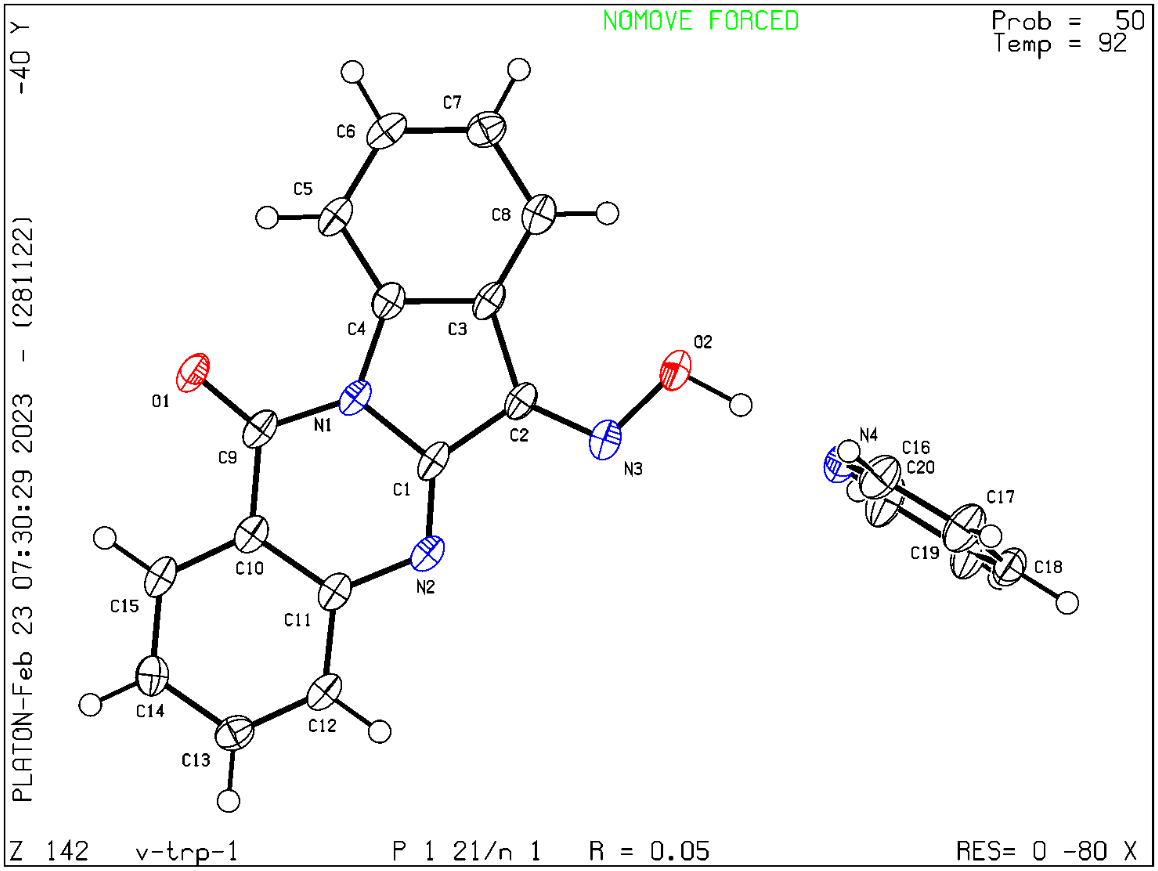

Supplement: Supplementary file 1 [file pharmaceutics-15-01802-s001.zip › 2249118_CheckCIF.pdf]
