# Supplementary material for: Experimental and Computational Investigation of the Oxime Bond Stereochemistry in c-Jun N-terminal Kinase 3 Inhibitors 11H-Indeno[1,2-b]quinoxalin-11-one Oxime and Tryptanthrin-6-oxime
Source: Pharmaceutics. 2023 Jun 23;15(7):1802. doi: 10.3390/pharmaceutics15071802 (PMC10383563; doi:10.3390/pharmaceutics15071802)
Supplement: Supplementary file 1 [file pharmaceutics-15-01802-s001.zip › Supplementary materials.pdf]

SUPPLEMENTARY MATERIALS

Experimental and computational investigation of the oxime bond stereochemistry in JNK3 kinase inhibitors IQ-1 and tryptanthrin-6-oxime

Vladislava V. Matveevskaya [1], Dmitry I. Pavlov [1], Anastasia R. Kovrizhina [2], Taisiya S. Sukhikh [1], Evgeniy H. Sadykov [1], Pavel V. Dorovatovskii [3], Vladimir A. Lazarenko [3], Andrei I. Khlebnikov [2,*] and Andrei S. Potapov [1,*]

[1] Nikolaev Institute of Inorganic Chemistry, Siberian Branch of the Russian Academy of Sciences, 3 Lavrentiev Ave., 630090, Novosibirsk, Russia; matveevskaya@niic.nsc.ru (V.V.M.), pavlov@niic.nsc.ru (D.I.P.), sukhikh@niic.nsc.ru (T.S.S.), sadykov@niic.nsc.ru (E.K.S.), potapov@niic.nsc.ru (A.S.P.)

[2] Kizhner Research Center, National Research Tomsk Polytechnic University, 30 Lenin Ave., 634050 Tomsk, Russia; ark4@tpu.ru (A.R.K.), aikhl@chem.org.ru (A.I.K.)

[3] National Research Centre "Kurchatov Institute", Kurchatov Square 1, Moscow 123182, Russia; vladimir.a.lazarenko@gmail.com (V.A.L.); paulgemini@mail.ru (P.V.D.)

* Correspondence: aikhl@tpu.ru (A.I.K.); potapov@niic.nsc.ru (A.S.P.)

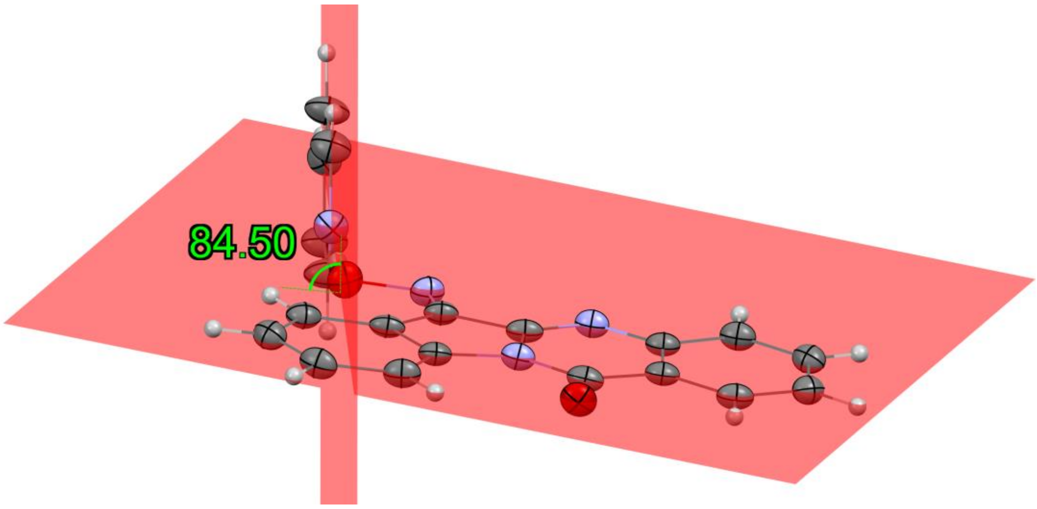

Figure S1. Fragment of the crystal structure of **Trp-Ox·Py** showing the relative orientation of pyridine and tryptanthrin rings.

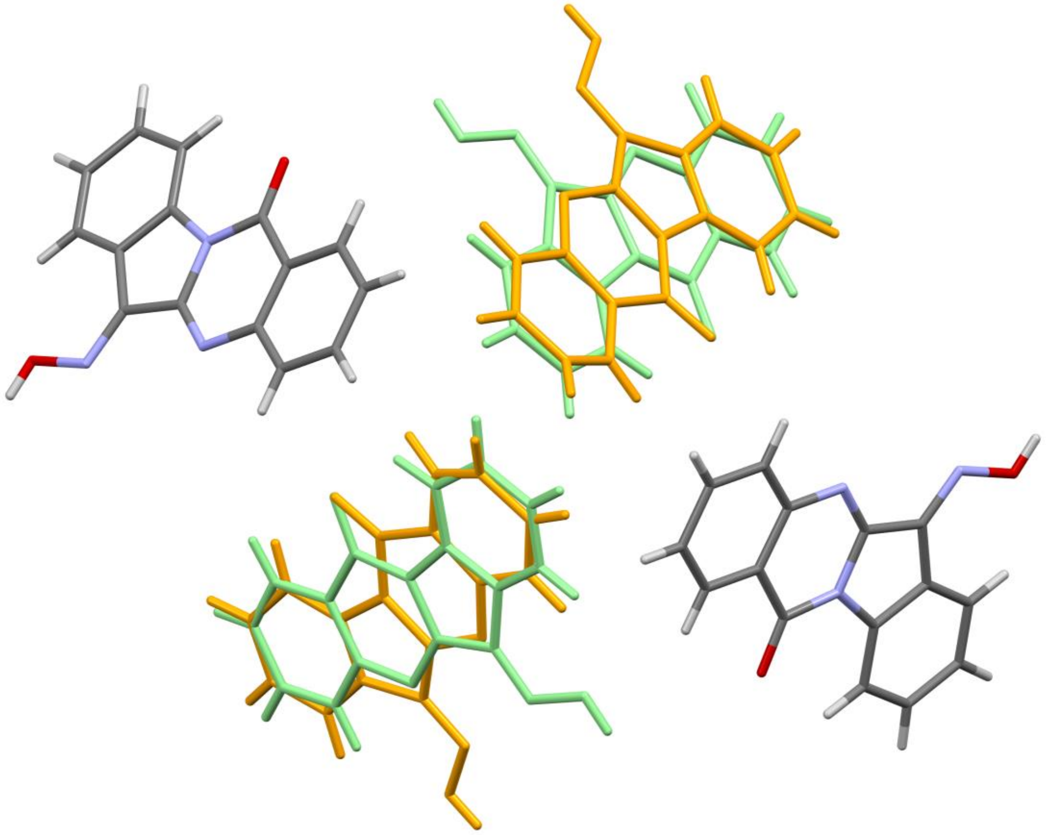

Figure S2. Crystal packing diagram of **Trp-Ox** showing two disordered positions of molecules (orange and green), view along axis b.

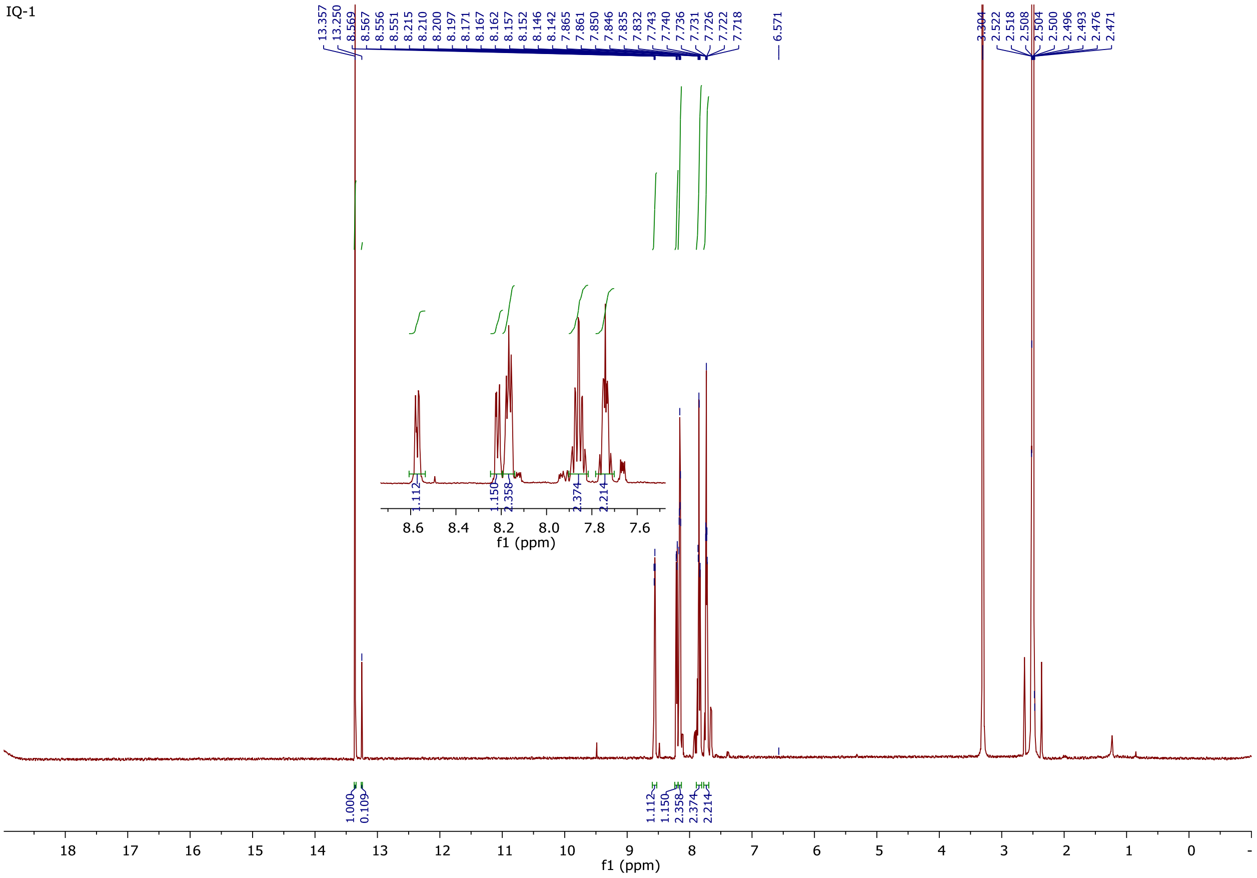

Figure S3. ^{1}H NMR spectrum of **IQ-1** in DMSO-d$_6$ at 500 MHz.

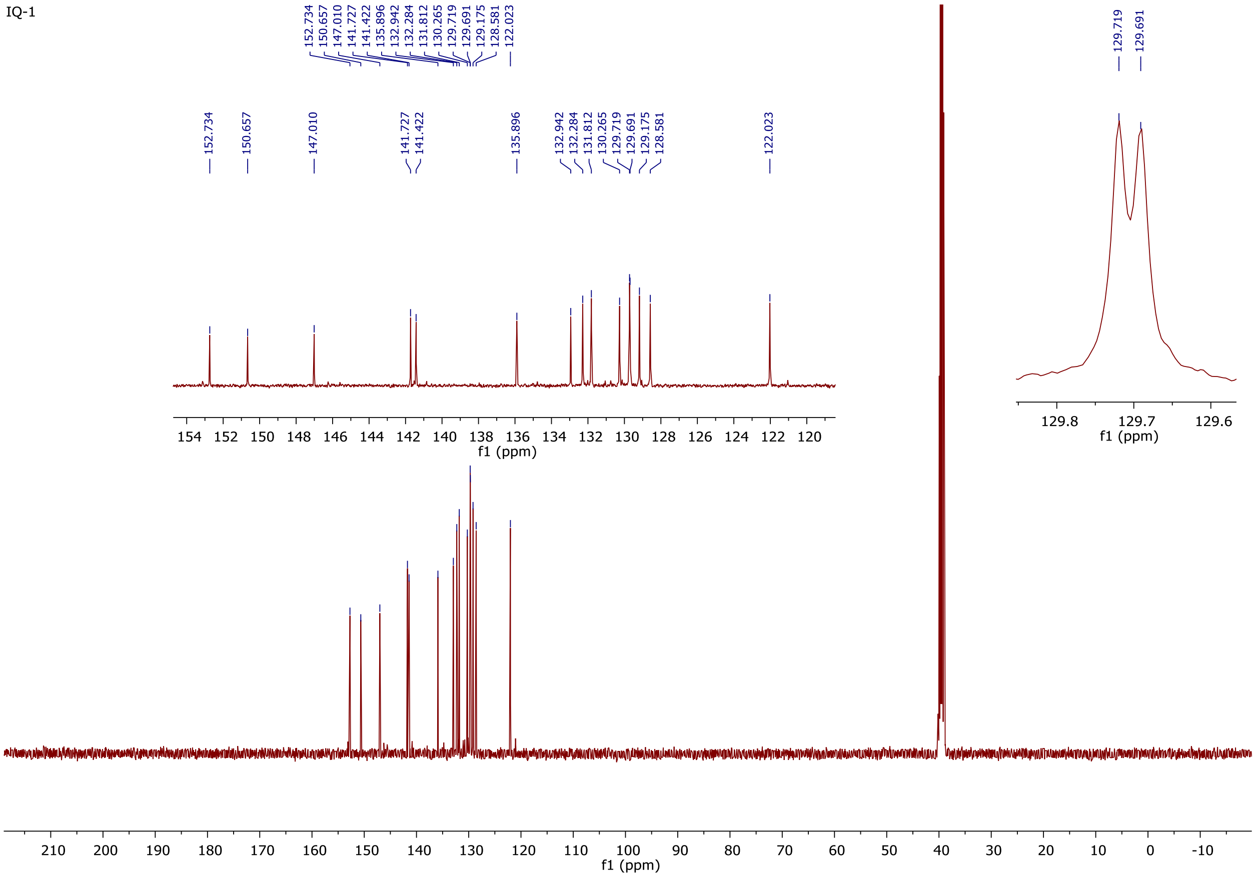

Figure S4. ^{13}C NMR spectrum of **IQ-1** in DMSO-d$_6$ at 125 MHz.

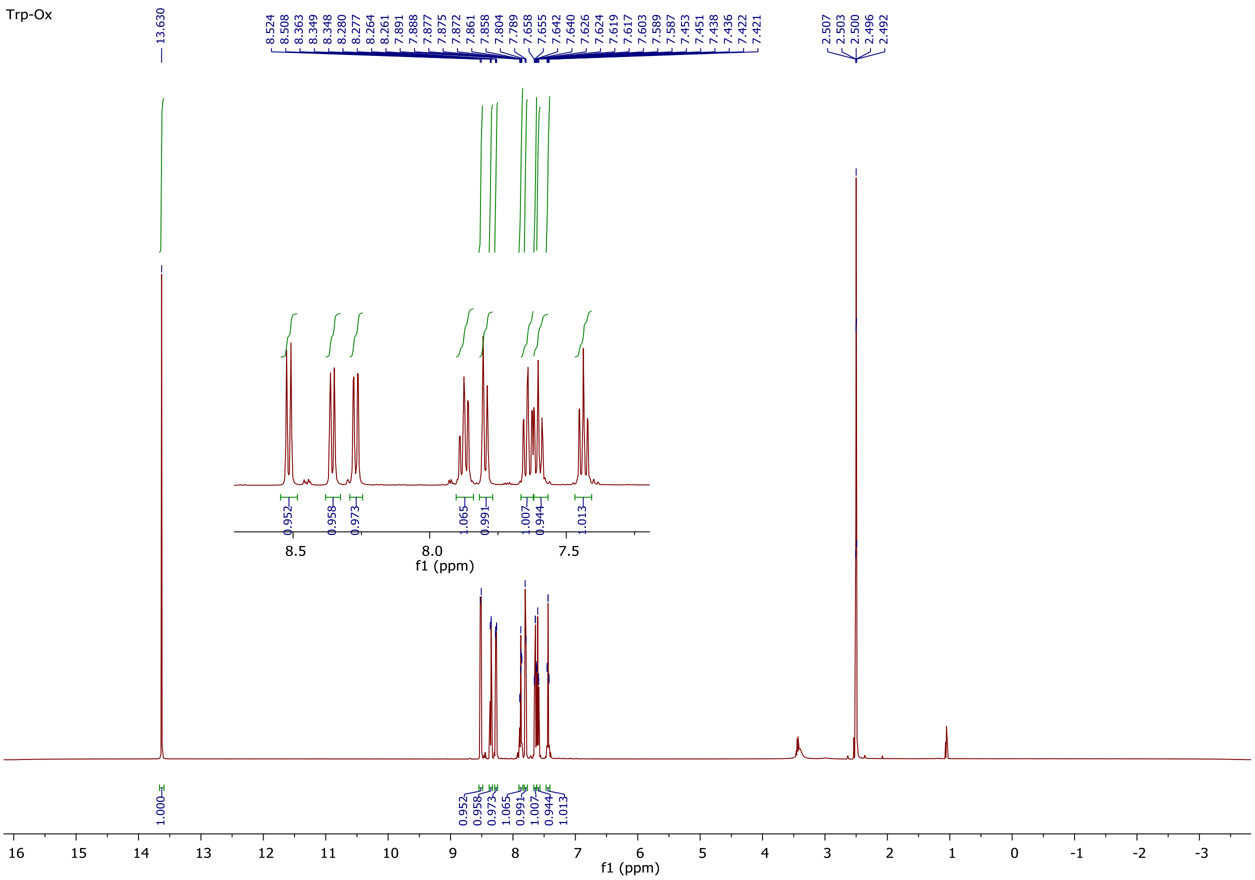

Figure S5. ^{1}H NMR spectrum of **Trp-Ox** in DMSO-d$_6$ at 500 MHz.

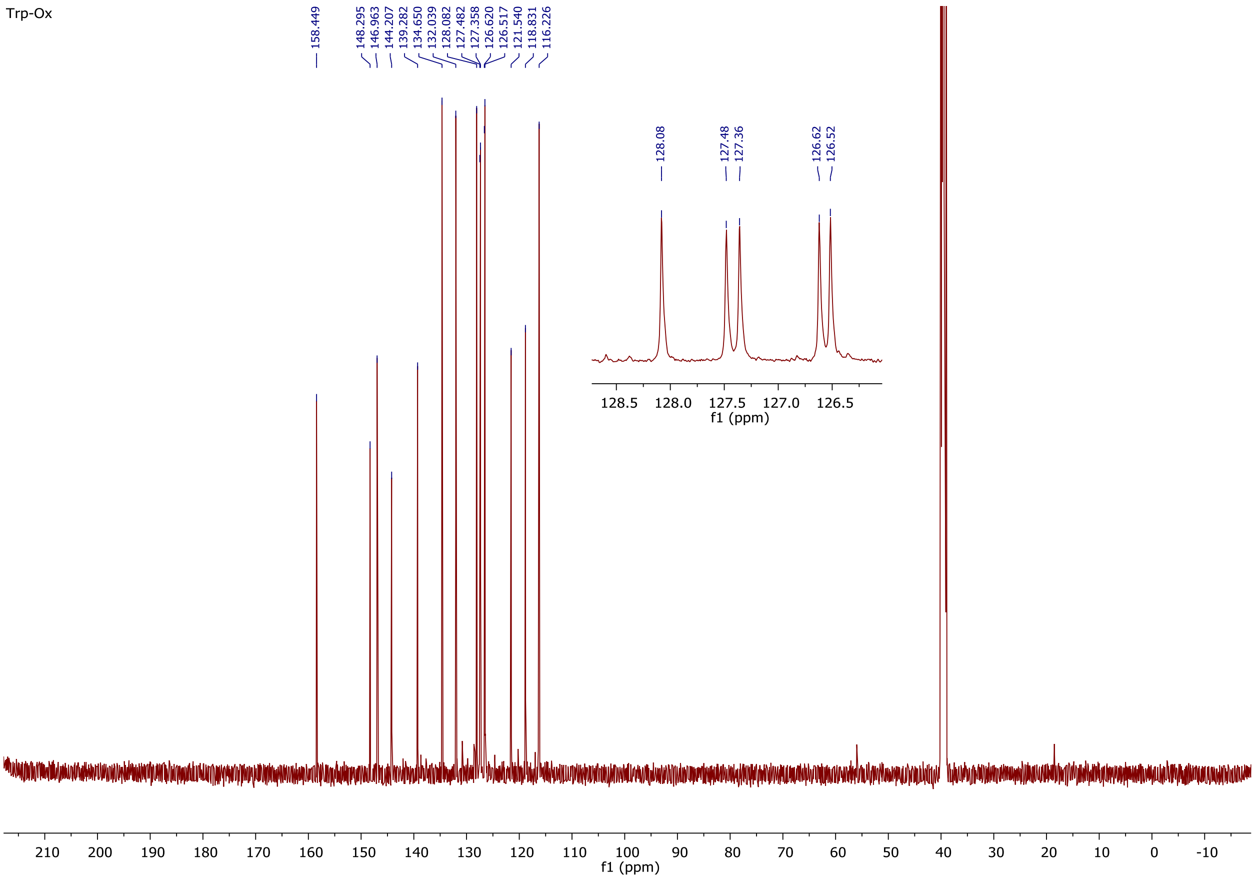

Figure S6. ^{13}C NMR spectrum of **Trp-Ox** in DMSO-d$_6$ at 125 MHz.

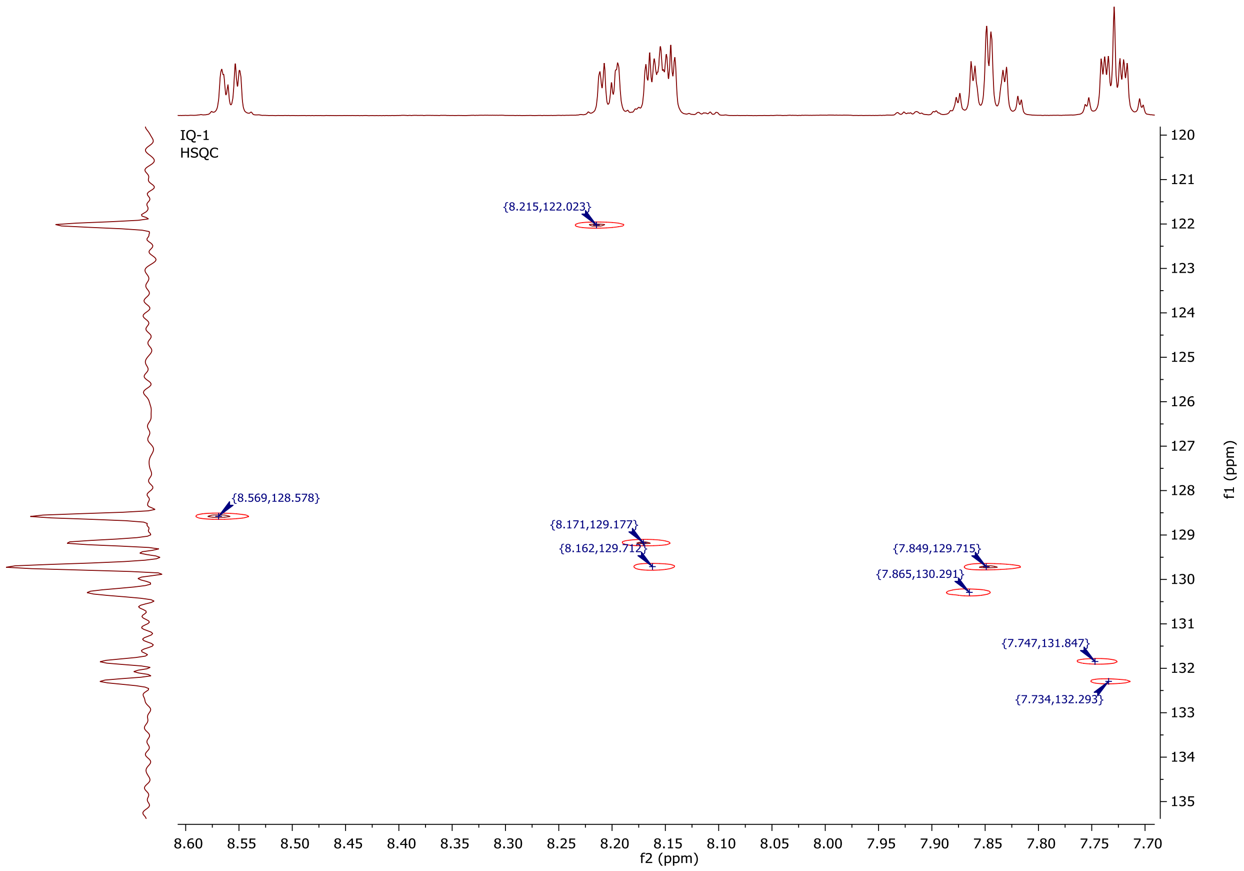

Figure S7. 2D HSQC ($^{1}H/^{13}C$) NMR spectrum of **IQ-1** in DMSO-d₆.

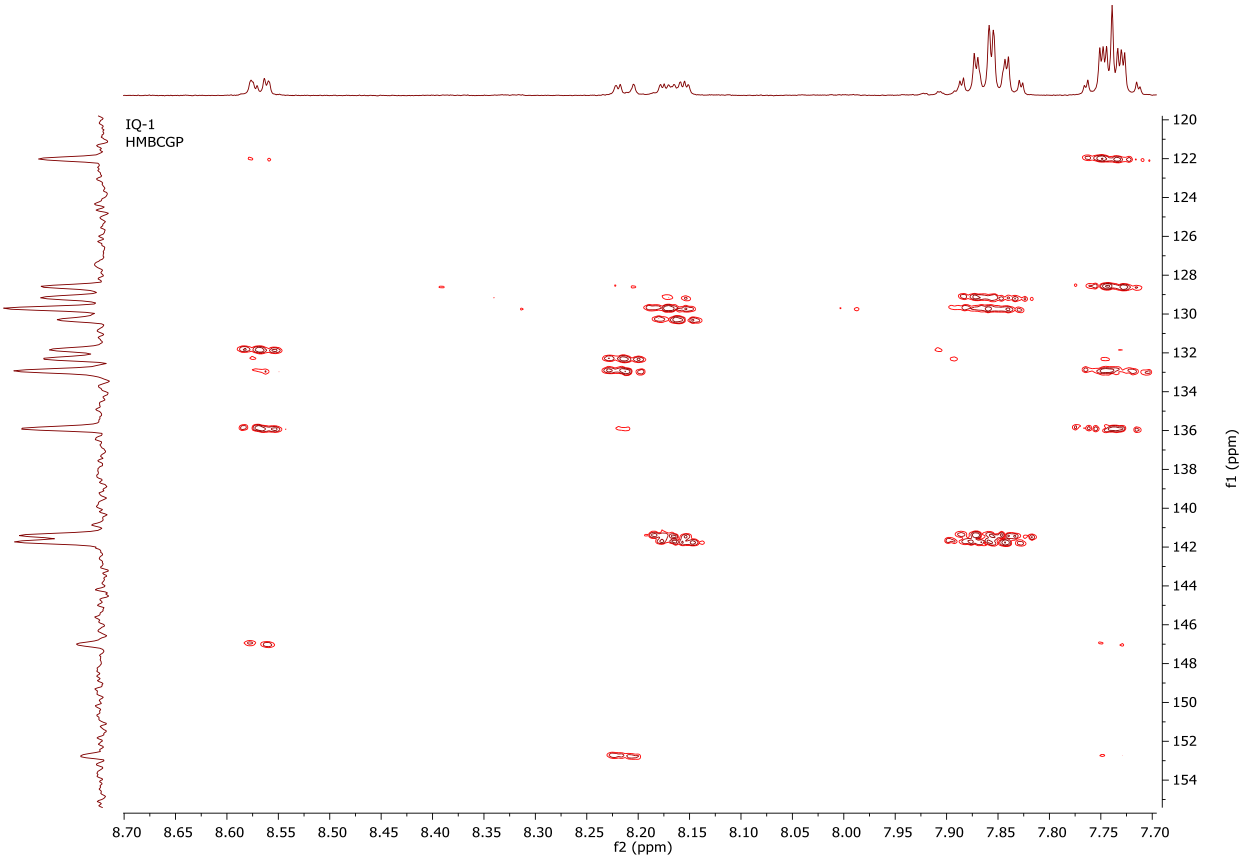

Figure S8. 2D HMBC ($^{1}H/^{13}C$) NMR spectrum of **IQ-1** in DMSO-d₆.

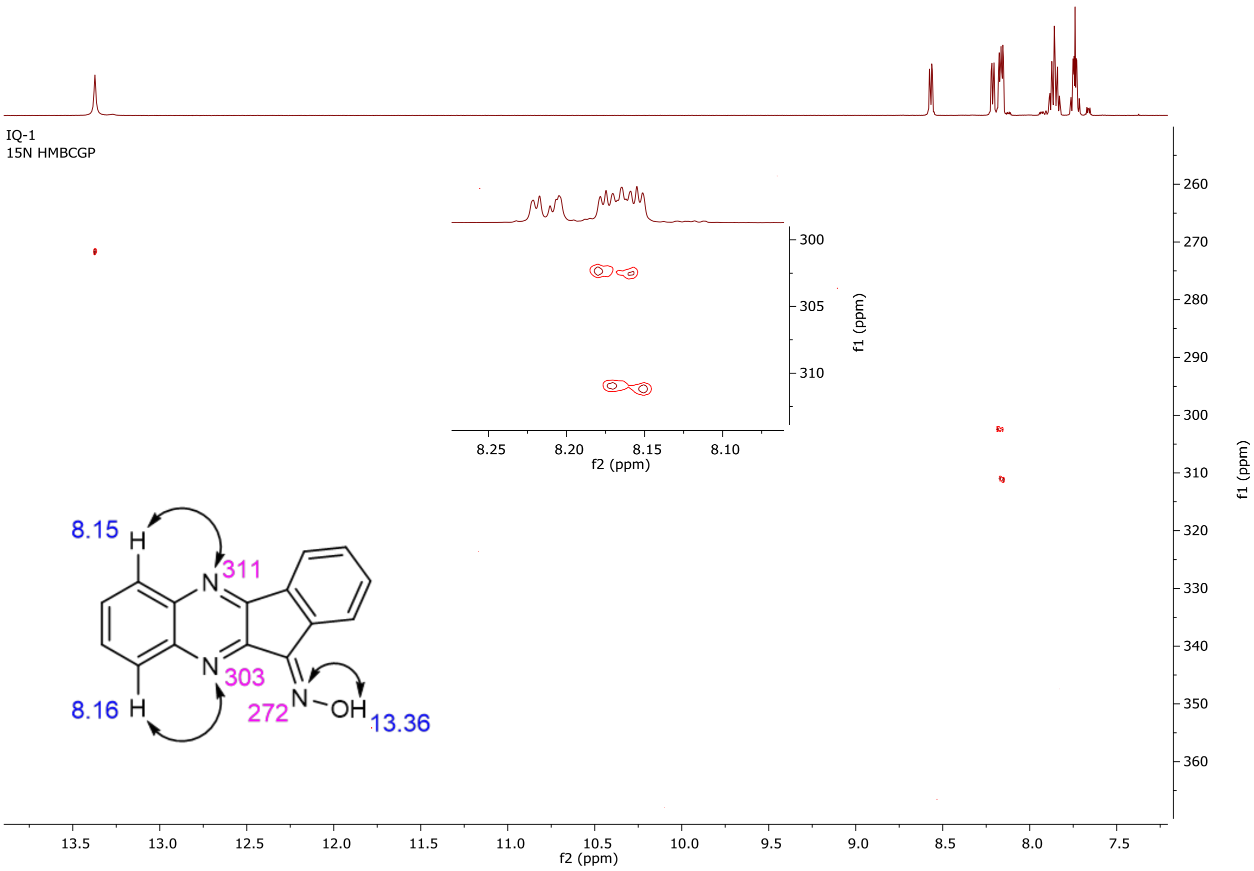

Figure S9. 2D HMBC ($^1H/^{15}N$) NMR spectrum of **IQ-1** in DMSO-d_6.

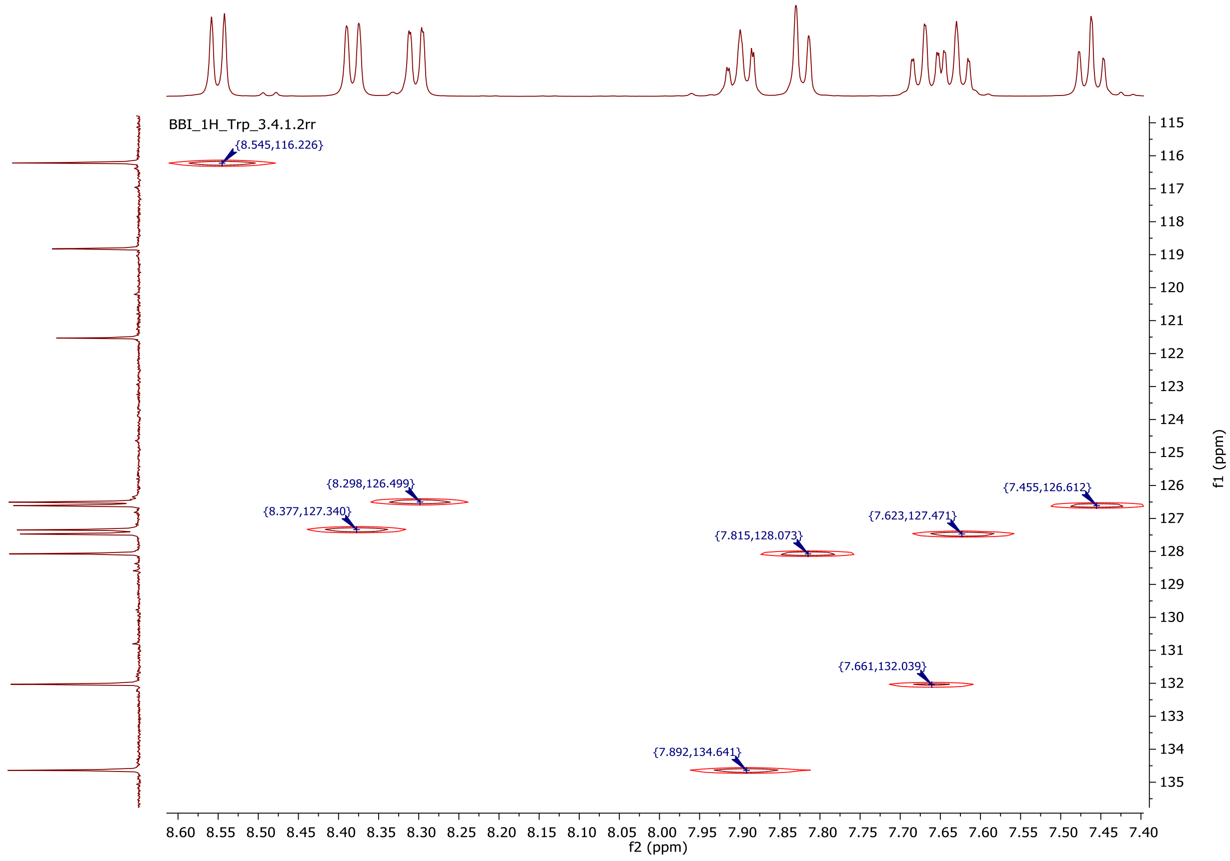

Figure S10. 2D HSQC ($^1H/^{13}C$) NMR spectrum of **Trp-Ox** in DMSO-d_6.

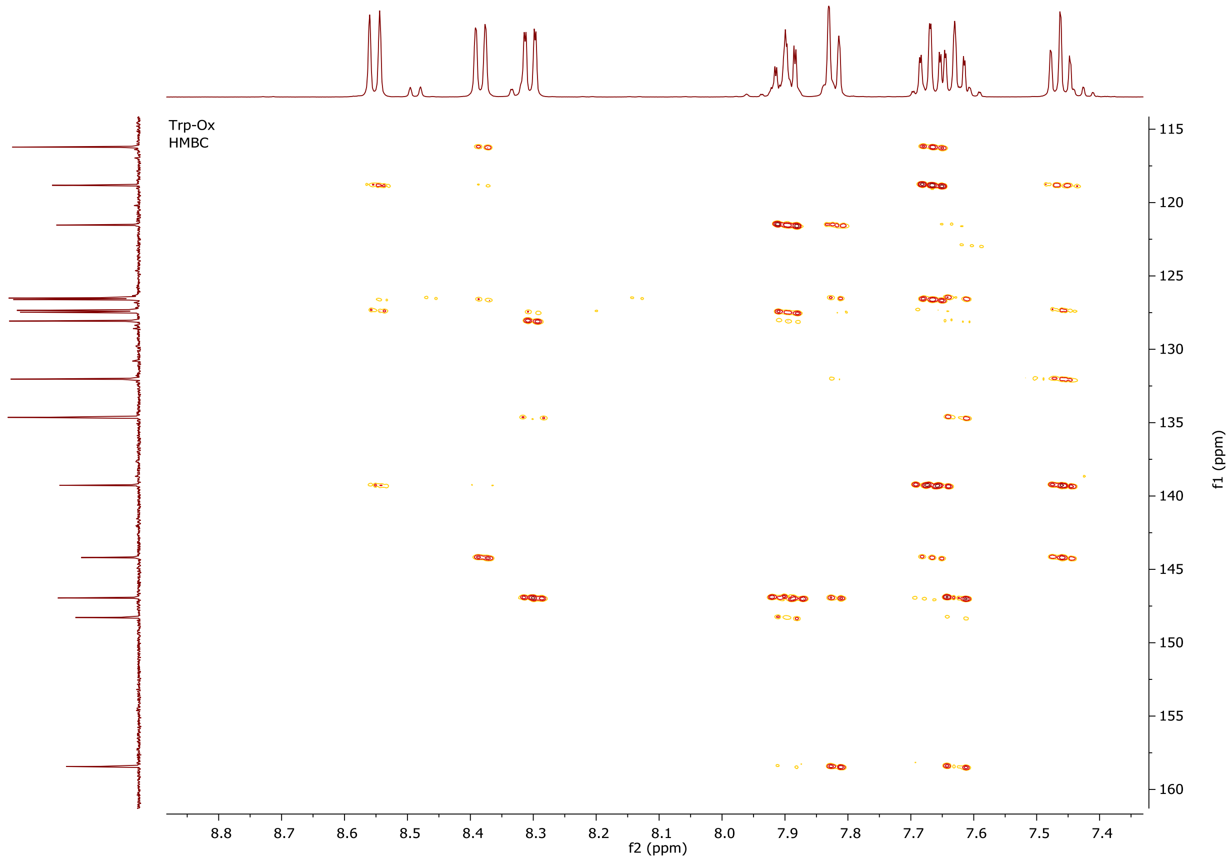

Figure S11. 2D HMBC ($^{1}H/^{13}C$) NMR spectrum of **Trp-Ox** in DMSO-d$_6$.

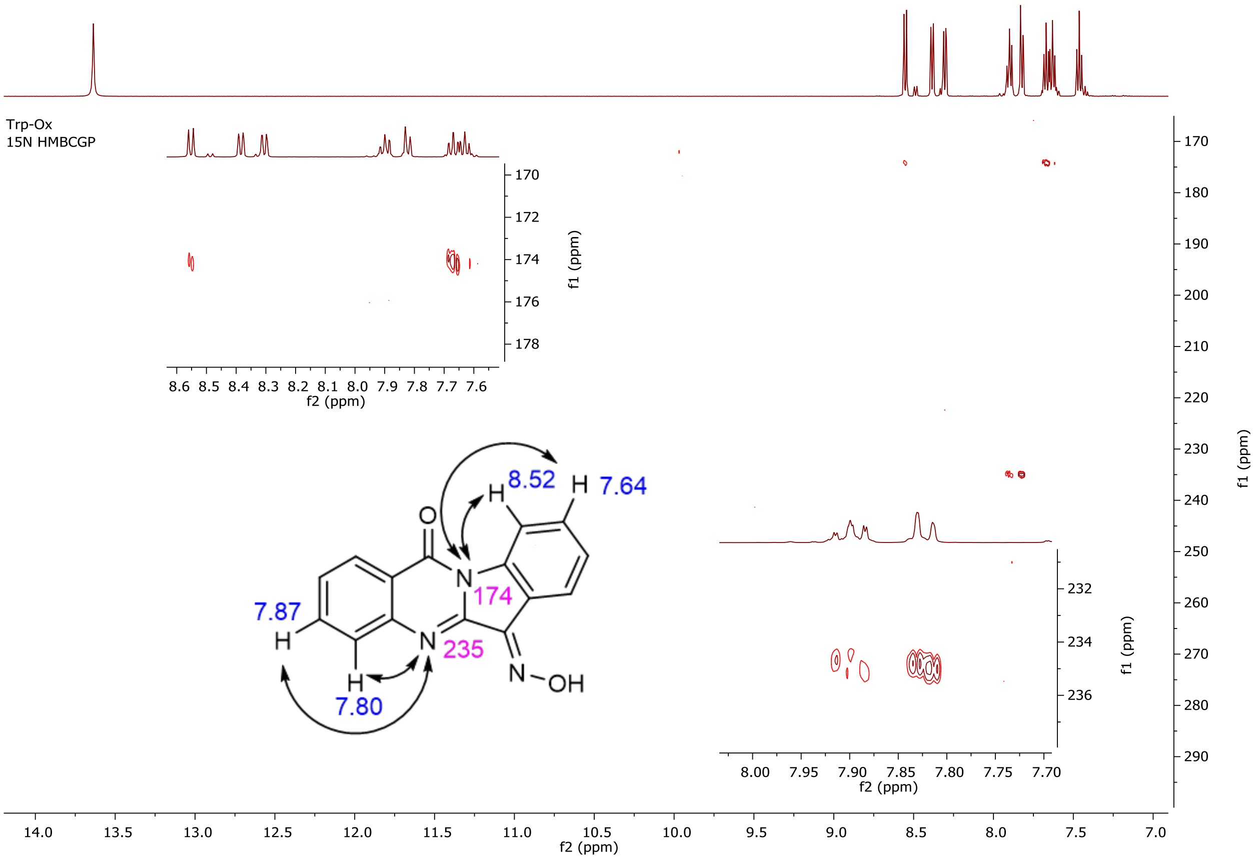

Figure S12. 2D HMBC ($^{1}H/^{15}N$) NMR spectrum of **Trp-Ox** in DMSO-d$_6$.

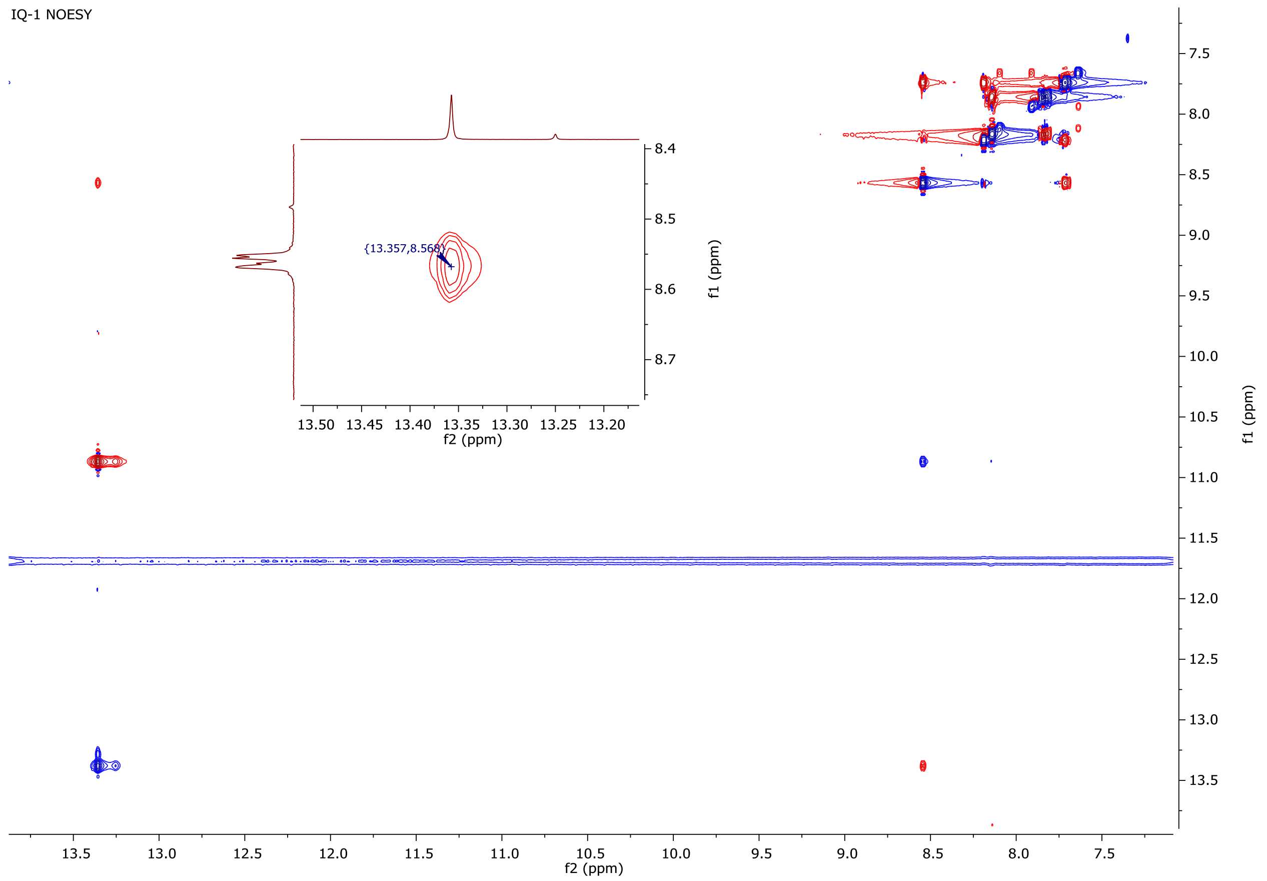

Figure S13. 2D NOESY ($^1H/^1H$) NMR spectrum of **IQ-1** in DMSO-d$_6$.

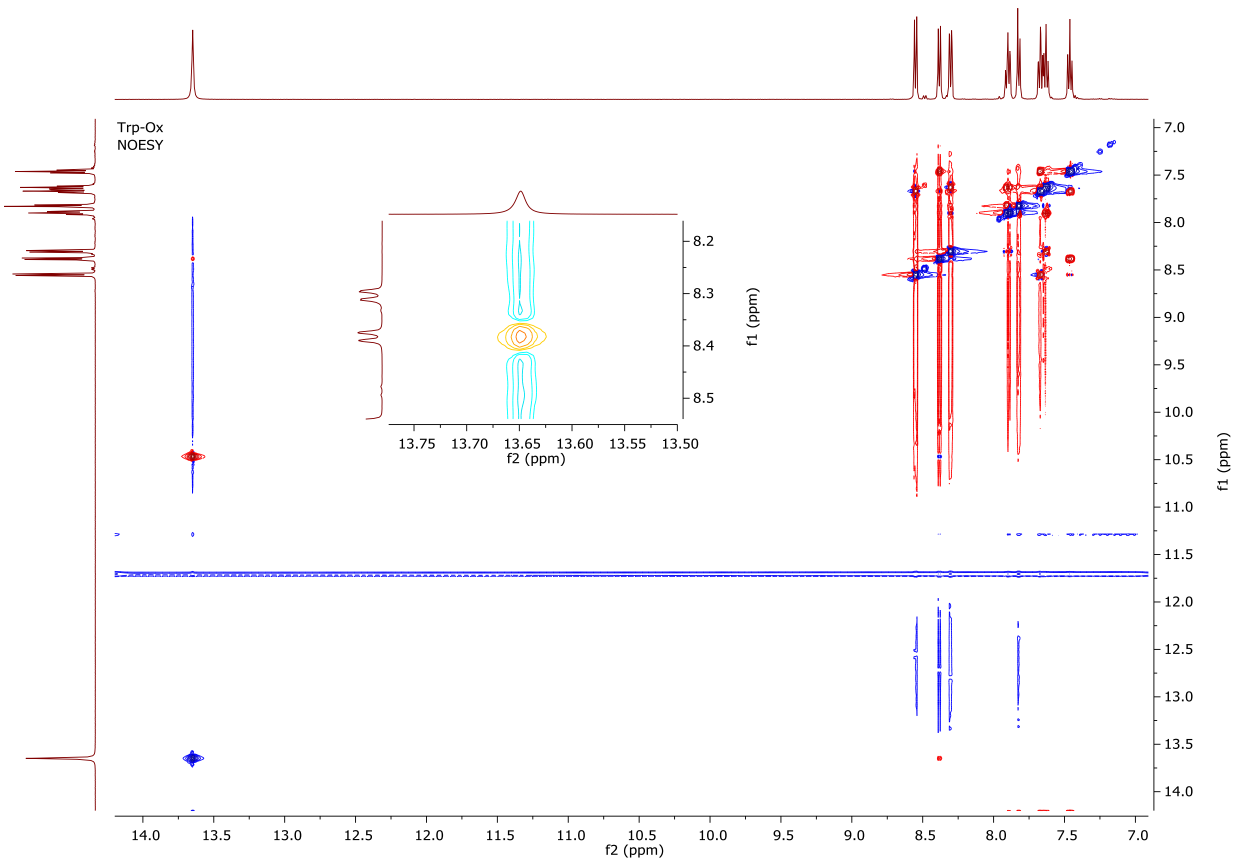

Figure S14. 2D NOESY ($^1H/^1H$) NMR spectrum of **Trp-Ox** in DMSO-d$_6$.

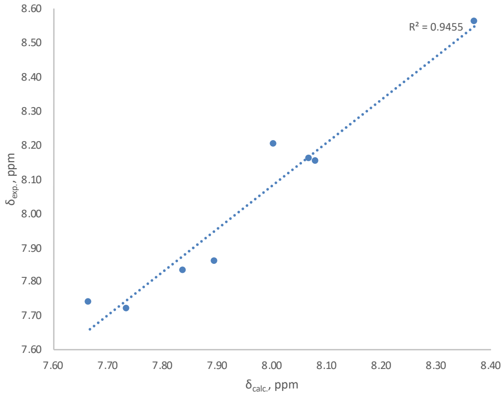

(**a**)

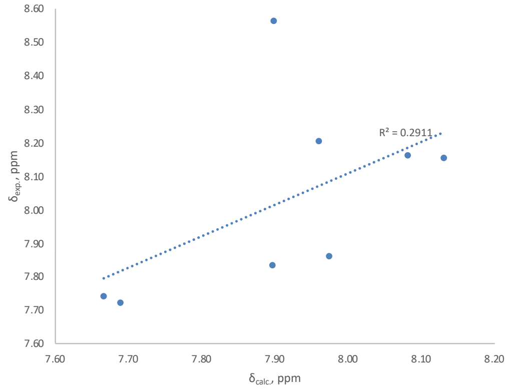

(**b**)

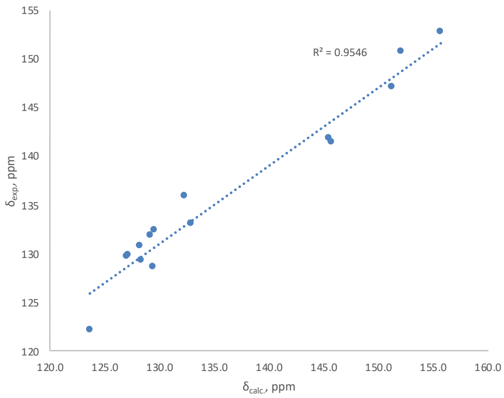

(**c**)

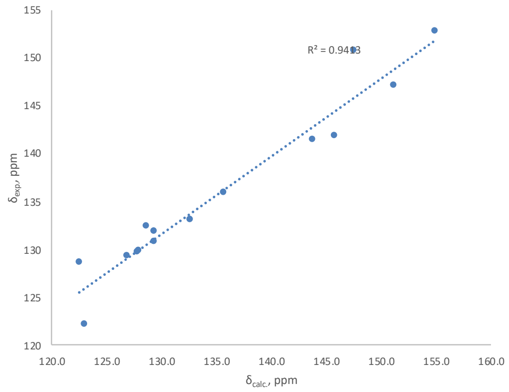

(**d**)

Figure S15. Correlation plots between the experimental and the calculated NMR chemical shifts (Method A) for: (**a**) *E*-**IQ-1**, ^{1}H; (**b**) *Z*-**IQ-1**, ^{1}H; (**c**) *E*-**IQ-1**, ^{13}C; (**d**) *Z*-**IQ-1**, ^{13}C.

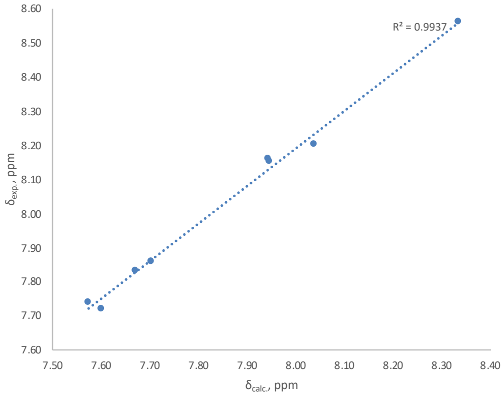

(**a**)

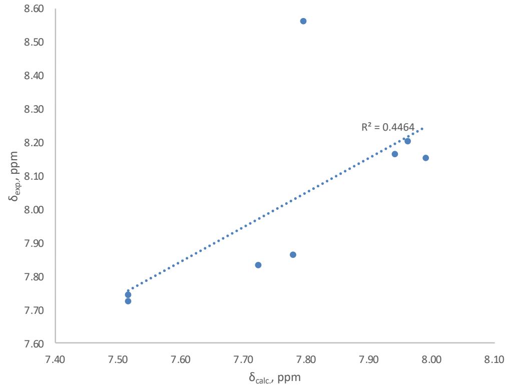

(**b**)

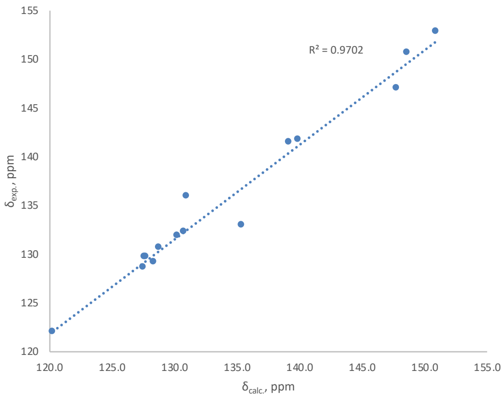

(**c**)

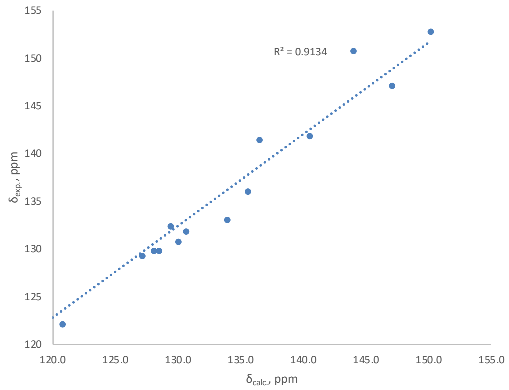

(**d**)

Figure S16. Correlation plots between the experimental and the calculated NMR chemical shifts (Method B) for: (**a**) *E*-**IQ-1**, ^{1}H; (**b**) *Z*-**IQ-1**, ^{1}H; (**c**) *E*-**IQ-1**, ^{13}C; (**d**) *Z*-**IQ-1**, ^{13}C.

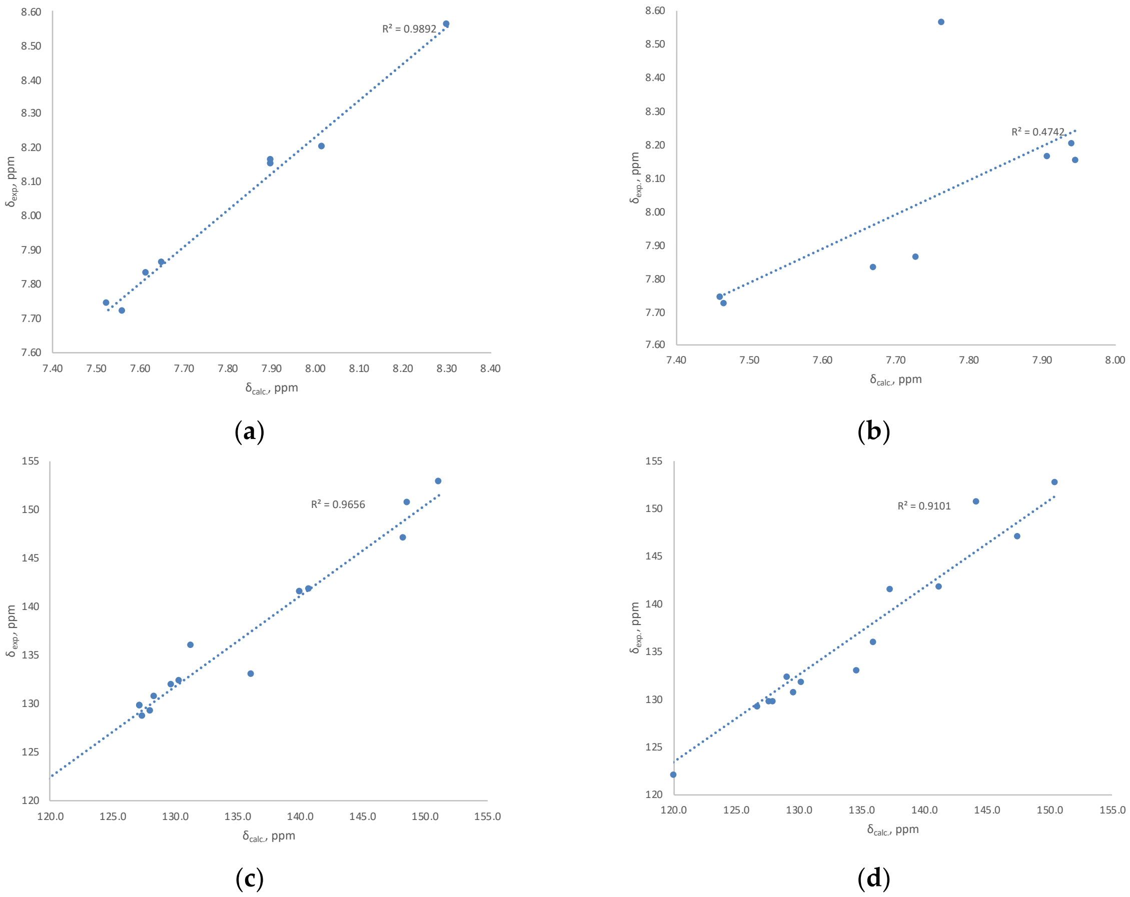

Figure S17. Correlation plots between the experimental and the calculated NMR chemical shifts (Method C) for: (**a**) *E*-**IQ-1**, ^{1}H; (**b**) *Z*-**IQ-1**, ^{1}H; (**c**) *E*-**IQ-1**, ^{13}C; (**d**) *Z*-**IQ-1**, ^{13}C.

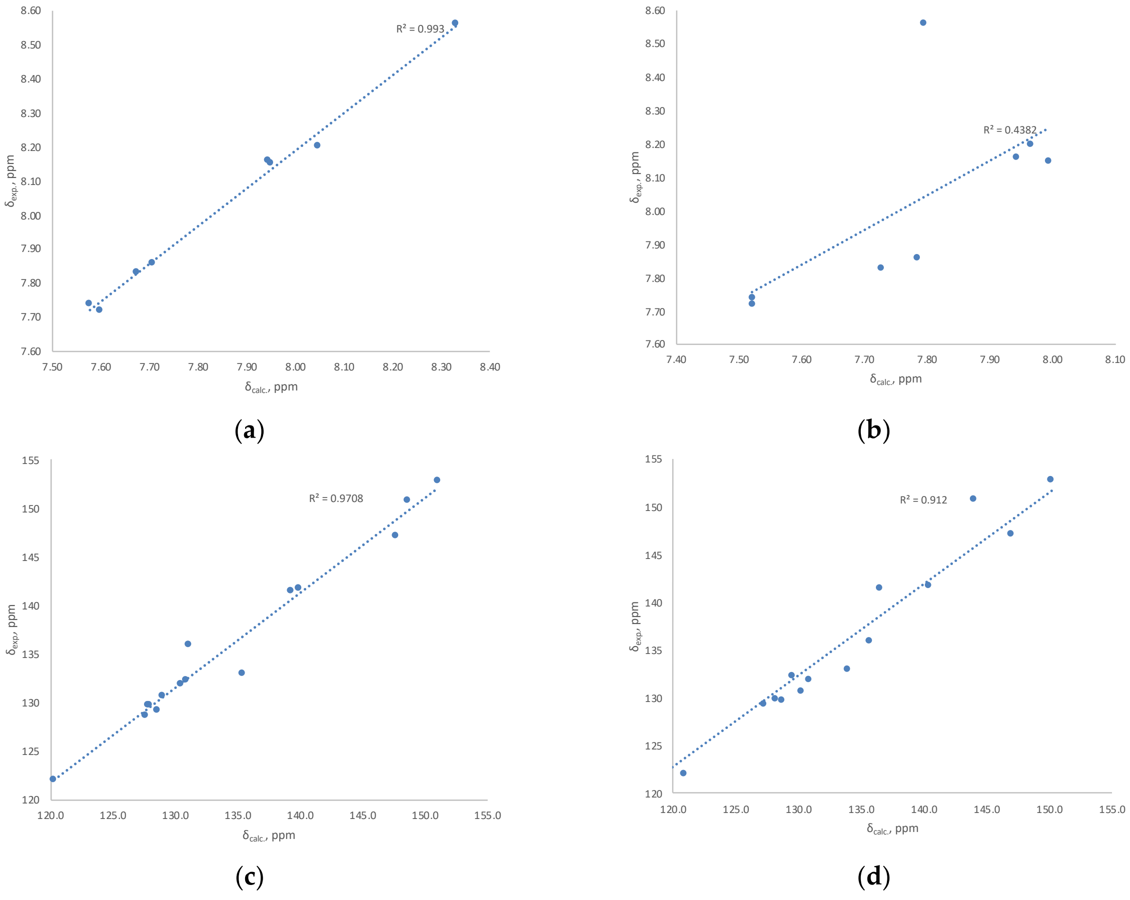

Figure S18. Correlation plots between the experimental and the calculated NMR chemical shifts (Method D) for: (**a**) *E*-**IQ-1**, ^{1}H; (**b**) *Z*-**IQ-1**, ^{1}H; (**c**) *E*-**IQ-1**, ^{13}C; (**d**) *Z*-**IQ-1**, ^{13}C.

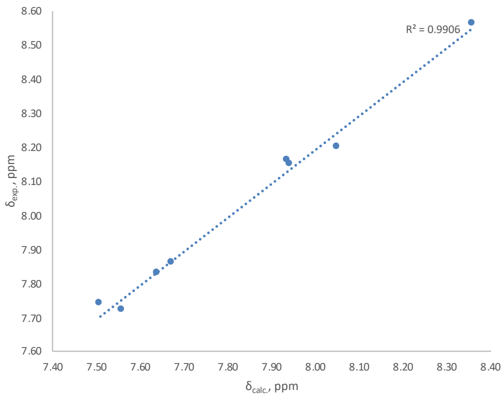

(**a**)

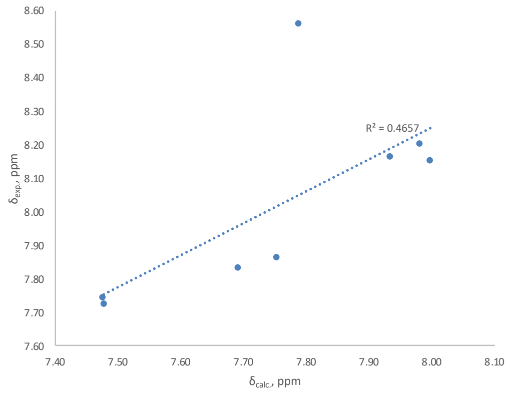

(**b**)

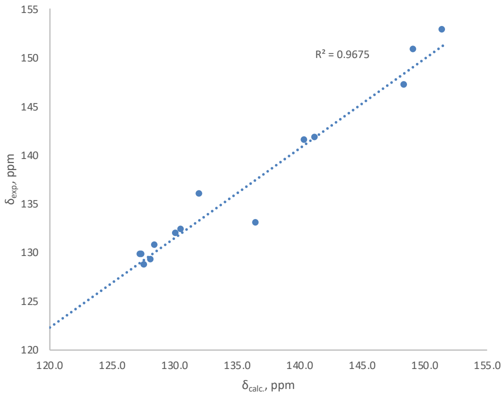

(**c**)

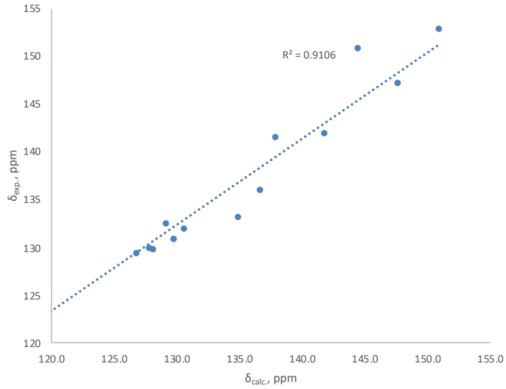

(**d**)

Figure S19. Correlation plots between the experimental and the calculated NMR chemical shifts (Method E) for: (**a**) *E*-**IQ-1**, ^{1}H; (**b**) *Z*-**IQ-1**, ^{1}H; (**c**) *E*-**IQ-1**, ^{13}C; (**d**) *Z*-**IQ-1**, ^{13}C.

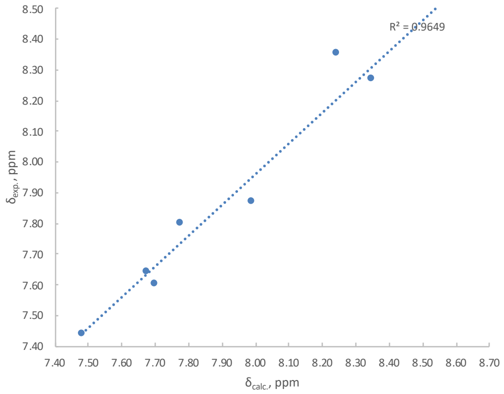

(**a**)

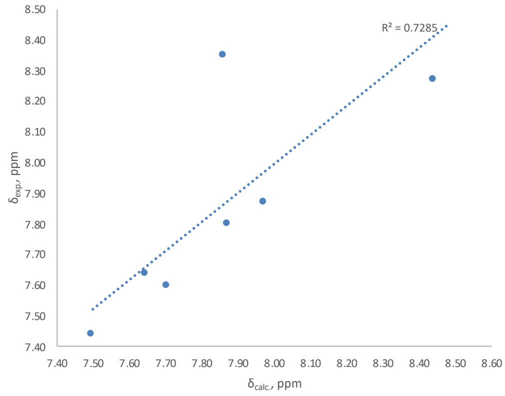

(**b**)

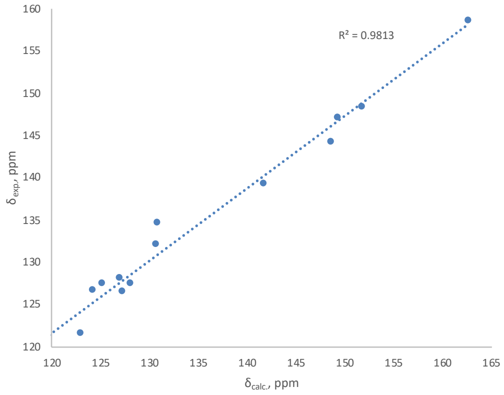

(**c**)

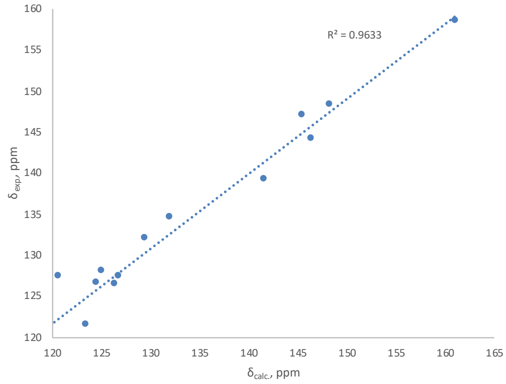

(**d**)

Figure S20. Correlation plots between the experimental and the calculated NMR chemical shifts (Method A) for: (**a**) *E*-**Trp-Ox**, ^{1}H; (**b**) *Z*-**Trp-Ox**, ^{1}H; (**c**) *E*-**Trp-Ox**, ^{13}C; (**d**) *Z*-**Trp-Ox**, ^{13}C.

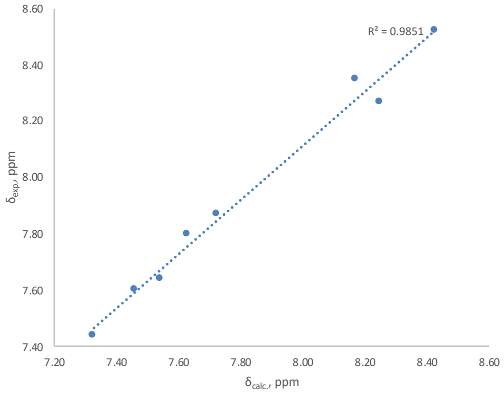

(**a**)

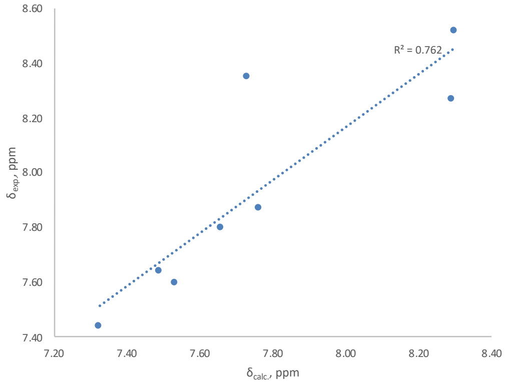

(**b**)

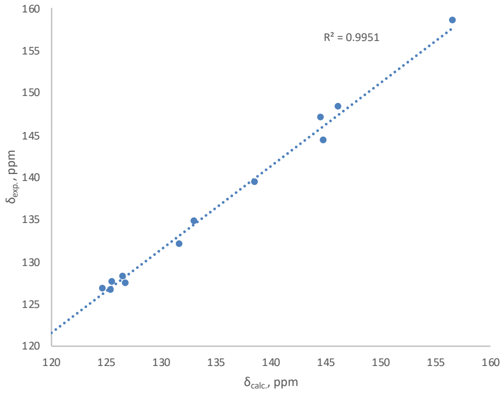

(**c**)

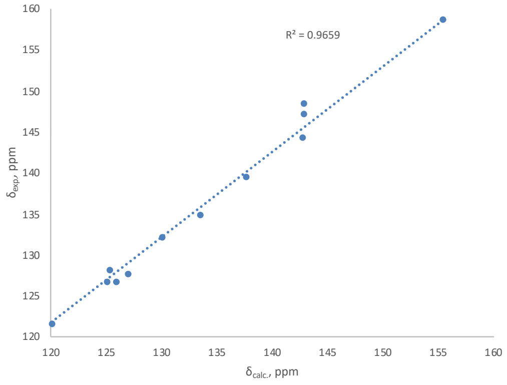

(**d**)

Figure S21. Correlation plots between the experimental and the calculated NMR chemical shifts (Method B) for: (**a**) *E*-**Trp-Ox**, ^{1}H; (**b**) *Z*-**Trp-Ox**, ^{1}H; (**c**) *E*-**Trp-Ox**, ^{13}C; (**d**) *Z*-**Trp-Ox**, ^{13}C.

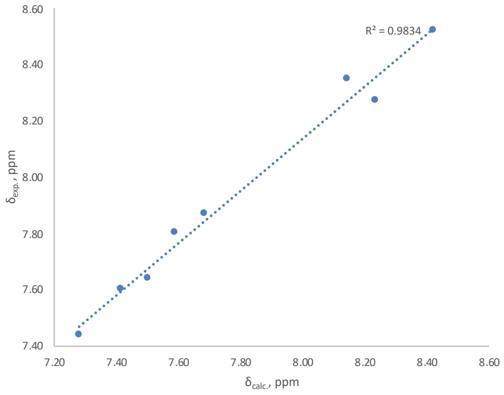

(**a**)

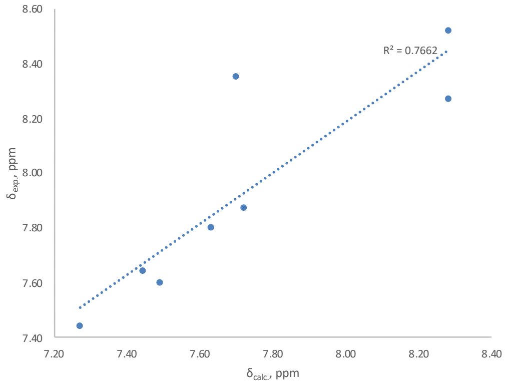

(**b**)

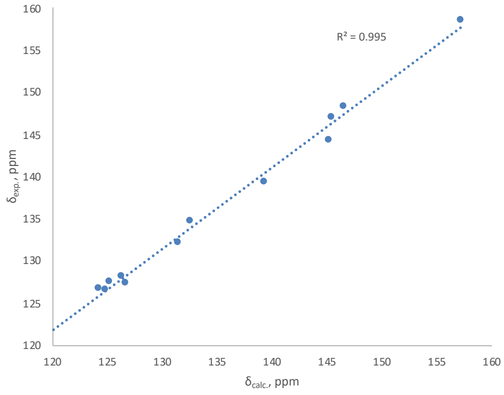

(**c**)

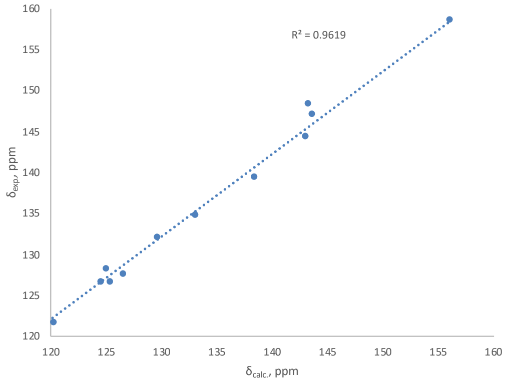

(**d**)

Figure S22. Correlation plots between the experimental and the calculated NMR chemical shifts (Method C) for: (**a**) *E*-**Trp-Ox**, ^{1}H; (**b**) *Z*-**Trp-Ox**, ^{1}H; (**c**) *E*-**Trp-Ox**, ^{13}C; (**d**) *Z*-**Trp-Ox**, ^{13}C.

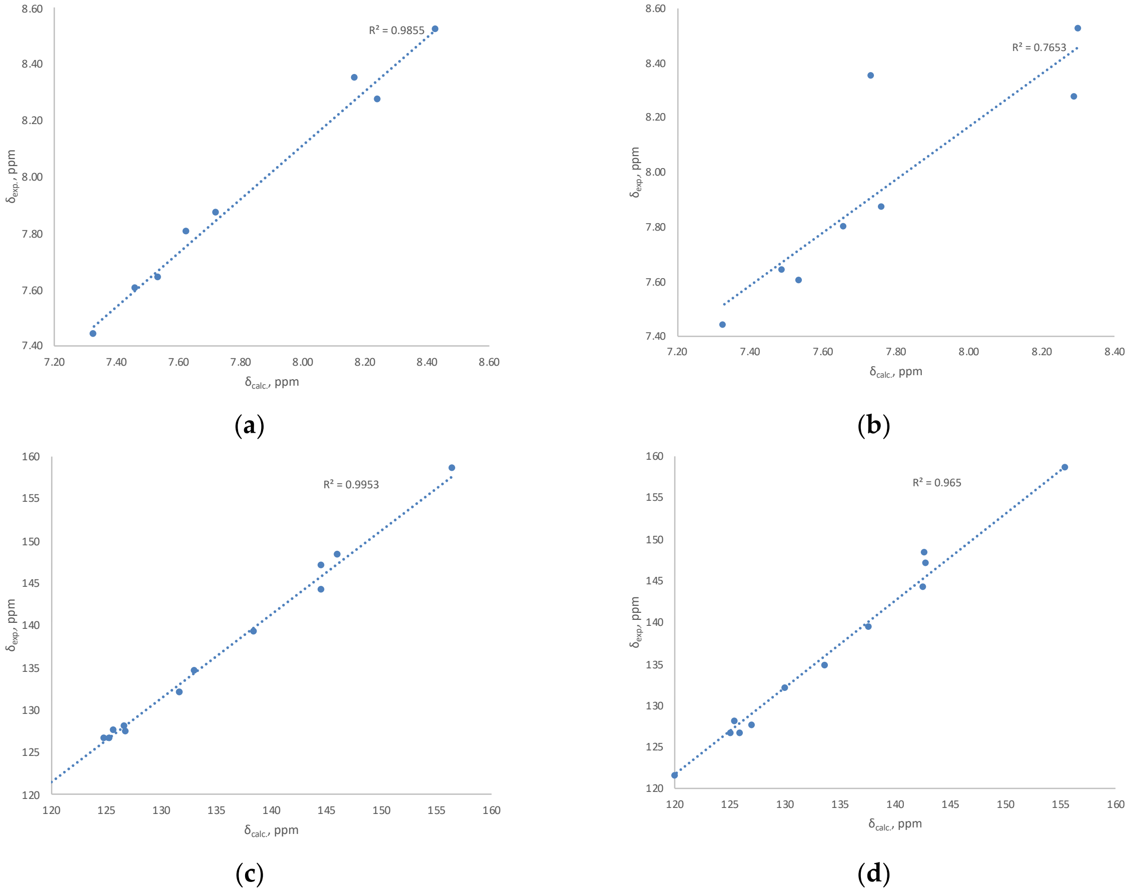

Figure S23. Correlation plots between the experimental and the calculated NMR chemical shifts (Method D) for: (**a**) *E*-**Trp-Ox**, ^{1}H; (**b**) *Z*-**Trp-Ox**, ^{1}H; (**c**) *E*-**Trp-Ox**, ^{13}C; (**d**) *Z*-**Trp-Ox**, ^{13}C.

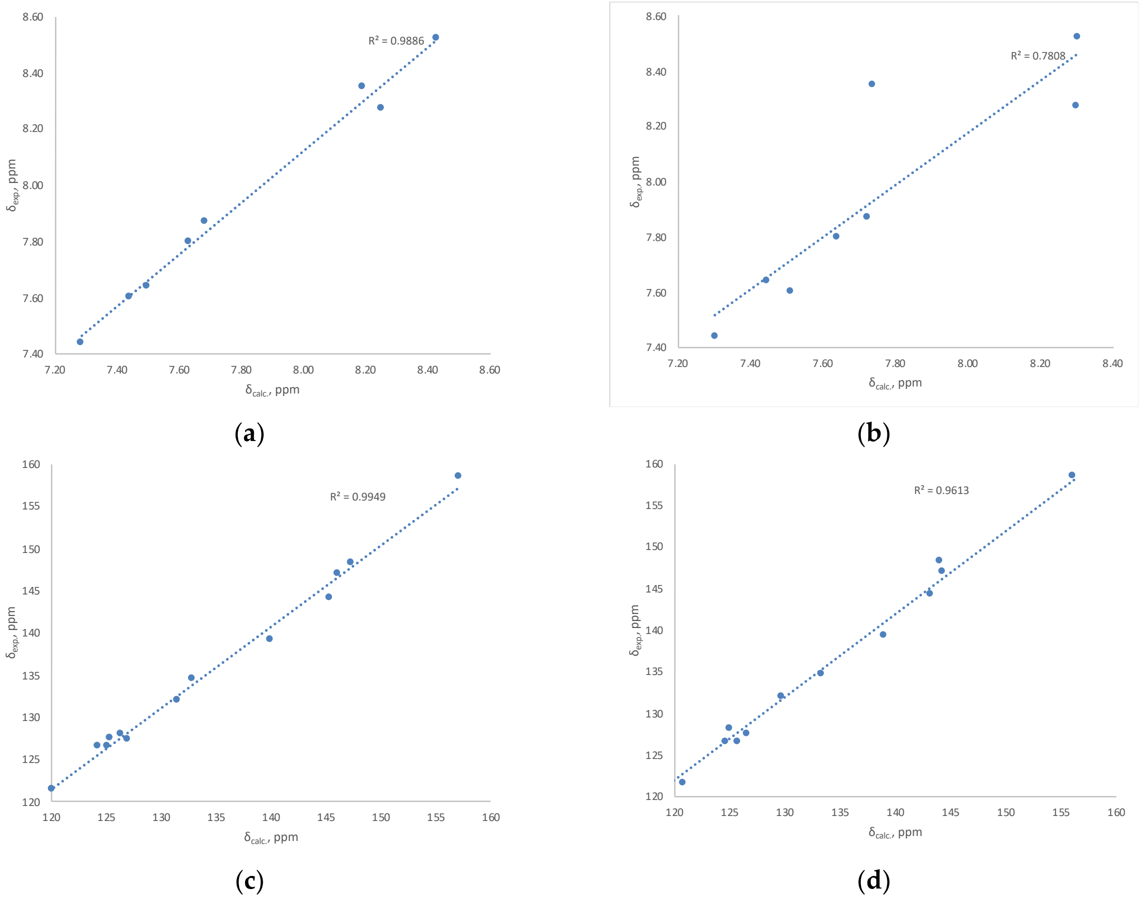

Figure S24. Correlation plots between the experimental and the calculated NMR chemical shifts (Method E) for: (**a**) *E*-**Trp-Ox**, ^{1}H; (**b**) *Z*-**Trp-Ox**, ^{1}H; (**c**) *E*-**Trp-Ox**, ^{13}C; (**d**) *Z*-**Trp-Ox**, ^{13}C.

Table S1. Calculated chemical shifts in NMR ^{1}H and ^{13}C spectra of E- and Z-isomers of **IQ-1** and experimental chemical shifts in DMSO-d_6.

**Atom label**	**Chemical shifts, ppm**					
	**Experimental in DMSO-d_6**	**Calculated using**				
		**Model A**	**Model B**	**Model C**	**Model D**	**Model E**
	**E-isomer**					
H-1 (d)	8.56	8.37	8.33	8.30	8.33	8.36
H-2 (t)	7.74	7.67	7.57	7.53	7.58	7.51
H-3 (t)	7.72	7.74	7.60	7.56	7.60	7.56
H-4 (d)	8.20	8.00	8.04	8.02	8.05	8.05
H-6 (d)	8.15	8.08	7.95	7.90	7.95	7.94
H-7 (t)	7.86	7.90	7.70	7.65	7.71	7.67
H-8 (t)	7.83	7.84	7.67	7.61	7.68	7.64
H-9 (d)	8.16	8.07	7.95	7.90	7.94	7.94
OH	13.37	8.37	7.77	7.74	7.77	7.79
C-1	128.58	129.5	127.6	127.4	127.6	127.7
C-1a	135.9	132.3	131.1	131.3	131.1	132.0
C-2	131.81	129.2	130.4	129.7	130.4	130.2
C-3	132.28	129.5	130.8	130.4	130.9	130.6
C-4	122.02	123.7	120.3	119.4	120.3	119.3
C-4a	132.94	132.9	135.4	136.1	135.4	136.6
C-4b	152.73	155.8	151.0	151.2	151.0	151.5
C-6	129.72	127.2	127.7	127.2	127.8	127.4
C-6a	141.73	145.5	140.0	140.7	139.9	141.3
C-7	130.66	128.2	128.9	128.3	128.9	128.6
C-8	129.69	127.0	127.8	127.2	127.9	127.4
C-9	129.18	128.3	128.4	128.0	128.5	128.2
C-9a	141.42	145.8	139.3	140.0	139.2	140.5
C-10a	150.66	152.1	148.6	148.6	148.6	149.2
C-11	147.01	151.3	147.8	148.3	147.7	148.5
	**Z-isomer**					
H-1 (d)	8.56	7.90	7.80	7.76	7.79	7.79
H-2 (t)	7.74	7.67	7.52	7.46	7.52	7.48
H-3 (t)	7.72	7.69	7.52	7.47	7.52	7.48
H-4 (d)	8.20	7.96	7.96	7.94	7.97	7.98
H-6 (d)	8.15	8.13	7.99	7.95	7.99	8.00
H-7 (t)	7.86	7.97	7.78	7.73	7.78	7.75
H-8 (t)	7.83	7.90	7.72	7.67	7.73	7.69
H-9 (d)	8.16	8.08	7.94	7.91	7.94	7.93
OH	13.37	13.45	12.92	13.00	12.90	13.11
C-1	128.58	122.5	119.7	118.9	119.6	119.2
C-1a	135.9	135.7	135.6	136.0	135.6	136.7
C-2	131.81	129.3	130.7	130.2	130.8	130.6
C-3	132.28	128.7	129.5	129.1	129.5	129.3
C-4	122.02	123.0	120.9	120.0	120.9	119.9
C-4a	132.94	132.6	134.0	134.6	133.9	135.0
C-4b	152.73	154.9	150.2	150.4	150.2	151.0
C-6	129.72	127.9	128.1	127.6	128.2	127.9
C-6a	141.73	145.8	140.5	141.2	140.4	141.9
C-7	130.66	129.4	130.1	129.6	130.2	129.8
C-8	129.69	127.8	128.6	128.0	128.7	128.2
C-9	129.18	126.8	127.2	126.8	127.3	126.9
C-9a	141.42	143.8	136.6	137.3	136.5	137.9
C-10a	150.66	147.6	144.0	144.2	144.0	144.5
C-11	147.01	151.2	147.1	147.4	147.0	147.7

Table S2. Calculated chemical shifts in NMR ^{1}H and ^{13}C spectra of E- and Z-isomers of **Trp-Ox** and experimental chemical shifts in DMSO-d_6.

Atom label	Chemical shifts, ppm					
	Experimental in DMSO-d_6	Calculated using Model A	Model B	Model C	Model D	Model E
	**E-isomer**					
H-1 (d)	8.27	8.35	8.25	8.23	8.25	8.25
H-2 (t)	7.60	7.70	7.46	7.41	7.46	7.44
H-3 (t)	7.87	7.99	7.72	7.68	7.72	7.68
H-4 (d)	7.80	7.78	7.63	7.59	7.63	7.63
H-7 (d)	8.35	8.24	8.17	8.14	8.17	8.19
H-8 (t)	7.44	7.48	7.33	7.28	7.33	7.29
H-9 (t)	7.64	7.68	7.54	7.50	7.54	7.50
H-10 (d)	8.52	8.60	8.43	8.42	8.43	8.43
OH	13.63	8.45	7.92	7.90	7.91	7.94
C-1	126.52	127.2	125.5	124.9	125.5	125.2
C-2	127.48	125.2	125.7	125.2	125.7	125.4
C-3	134.65	130.9	133.1	132.6	133.1	132.9
C-4	128.08	127.0	126.7	126.3	126.8	126.4
C-4a	146.96	149.3	144.7	145.4	144.6	146.1
C-5a	148.3	151.8	146.3	146.5	146.2	147.3
C-6	144.21	148.6	144.8	145.2	144.6	145.4
C-6a	118.83	117.0	116.5	116.7	116.4	117.4
C-7	127.36	128.1	126.9	126.7	126.9	127.0
C-8	126.62	124.2	124.8	124.3	124.9	124.3
C-9	132.04	130.6	131.8	131.4	131.8	131.5
C-10	116.23	115.8	115.0	114.5	115.0	114.3
C-10a	139.28	141.7	138.7	139.3	138.6	140.0
C-12	158.45	162.7	156.7	157.2	156.6	157.2
C-12a	121.54	123.0	119.4	119.5	119.4	120.2
	**Z-isomer**					
H-1 (d)	8.27	8.44	8.29	8.28	8.29	8.30
H-2 (t)	7.60	7.70	7.53	7.49	7.53	7.51
H-3 (t)	7.87	7.97	7.76	7.72	7.76	7.72
H-4 (d)	7.80	7.87	7.66	7.63	7.66	7.64
H-7 (d)	8.35	7.86	7.73	7.70	7.73	7.74
H-8 (t)	7.44	7.50	7.32	7.27	7.33	7.30
H-9 (t)	7.64	7.64	7.49	7.45	7.49	7.45
H-10 (d)	8.52	8.48	8.30	8.28	8.30	8.30
OH	13.63	14.25	13.64	13.73	13.63	13.92
C-1	126.52	126.5	126.0	125.4	126.0	125.8
C-2	127.48	126.8	127.1	126.6	127.1	126.6
C-3	134.65	132.1	133.6	133.1	133.7	133.3
C-4	128.08	125.1	125.4	125.0	125.6	125.0
C-4a	146.96	145.5	142.9	143.7	142.8	144.3
C-5a	148.3	148.2	142.9	143.3	142.8	144.1
C-6	144.21	146.4	142.8	143.1	142.7	143.3
C-6a	118.83	120.3	119.9	120.3	119.9	121.1
C-7	127.36	120.6	119.2	118.4	119.1	118.7
C-8	126.62	124.5	125.1	124.6	125.2	124.8
C-9	132.04	129.4	130.1	129.7	130.1	129.8
C-10	116.23	115.9	115.2	114.8	115.2	114.6
C-10a	139.28	141.6	137.8	138.4	137.7	139.0
C-12	158.45	161.1	155.6	156.1	155.5	156.2
C-12a	121.54	123.5	120.2	120.3	120.2	120.9
